# Supplementary material for: Link Worker social prescribing to improve health and well-being for people with long-term conditions: qualitative study of service user perceptions
Source: BMJ Open. 2017 Jul 16;7(7):e015203. doi: 10.1136/bmjopen-2016-015203 (PMC5541496; doi:10.1136/bmjopen-2016-015203)
Supplement: Author's manuscript [file bmjopen-2016-015203.draft_revisions.pdf]

BMJ Open

Social prescribing to improve health and wellbeing for people with long-term conditions: qualitative study of service user perceptions

Journal:	*BMJ Open*
Manuscript ID	bmjopen-2016-015203
Article Type:	Research
Date Submitted by the Author:	15-Nov-2016
Complete List of Authors:	Moffatt, Suzanne; Newcastle University, Institute of Health and Society<br>Steer, Mel; Newcastle University, Institute of Health and Society<br>Lawson, Sarah; Newcastle University, Institute of Health and Society<br>Penn, Linda; Newcastle University, Institute of Health and Society<br>O'Brien, Nicola; Newcastle University, Institute of Health and Society
**Primary Subject Heading**:	Public health
Secondary Subject Heading:	Qualitative research
Keywords:	Social prescribing, Long-term condition management, Health inequalities, Multi-morbidity

SCHOLARONE™
Manuscripts

For peer review only

Social prescribing to improve health and wellbeing for people with long-term conditions: qualitative study of service user perceptions.

Suzanne Moffatt, PhD[1]

M Steer, PhD

S Lawson

L Penn, PhD

N O'Brien, PhD

Word count: 3477 (excluding table, quotes and references)

Word count:5584 (including table and quotes, excluding references)

1.Corresponding author

Suzanne Moffatt

Senior Lecturer in Social Gerontology

Institute of Health and Society

Newcastle University

Campus for Ageing and Vitality

Biomedical Research Building

Newcastle upon Tyne, NE4 5PL

Email: suzanne.moffatt@ncl.ac.uk

Tel +44 (0) 191 208 5005

For peer review only - http://bmjopen.bmj.com/site/about/guidelines.xhtml

For peer review only

ABSTRACT

Objectives: To describe the experiences of primary care patients with long- term conditions who are referred to and engage with a social prescribing intervention and identify the impact of the intervention on health and wellbeing.

Design: Qualitative study using semi-structured interviews incorporating a thematic analysis of the data.

Intervention: Social prescribing comprising personalised support to identify meaningful health and wellness goals, and ongoing support to achieve agreed objectives together with linkage into appropriate services, facilitated by a Link Worker.

Setting: Inner-city area in West Newcastle upon Tyne, UK (population N=132,000) ranked 40th most socio-economically deprived in England, served by 18 general practices.

Participants: Thirty adults, 14 female, 16 male aged 40-74, mean age 62 years, 24 White British, 1 White Irish, five from Black and Minority Ethnic communities, with long term conditions.

Results: Most participants experienced multi- morbidity combined with mental health problems, low self-confidence and social isolation; and all were adversely affected physically, emotionally and socially by their health problems. The intervention engendered feelings of control and self- confidence, reduced social isolation and had a positive impact on health-related behaviours including weight loss, healthier eating and increased physical activity. Management of long-term conditions and mental health in the face of multi-morbidity improved and participants reported greater resilience and more effective problem solving strategies.

For peer review only - http://bmjopen.bmj.com/site/about/guidelines.xhtml

Conclusions: Findings suggest that tackling complex and long- term health problems requires an extensive holistic approach not possible in routine primary care. This model of social prescribing, which takes into account physical, mental health, social and economic issues, was successful for patients who engaged with the service. Future research on a larger scale is required to assess when and for whom social prescribing is clinically and cost effective.

For peer review only

For peer review only - http://bmjopen.bmj.com/site/about/guidelines.xhtml

Strengths and limitations of this study

- This is the first UK study to provide detailed insight into the impact of social prescribing on health and wellbeing among adults with long-term conditions living in an area of high socio-economic deprivation.
- Our interview data show that among patients willing to engage, the social prescribing intervention engendered feelings of control and self- confidence, reduced social isolation and led to positive physical changes such as weight loss, increased physical activity, improved long-term condition management and mental health, greater resilience and effective coping strategies to manage relapses.
- Key elements of the social prescribing model are that it: addresses the co-existence of multi-morbidity, mental health problems and social isolation; is long-term in nature; and, where applicable, tackles related socio-economic issues.
- A strength of this work is the depth and consistency of participant accounts regarding the impact of the intervention. A limitation is that we did not capture the experiences of patients with long-term conditions who refuse a referral or who drop out of the programme early.
- Our study adds to the emerging evidence base on social prescribing by demonstrating improvements in health-related behaviours and long-term condition management.

For peer review only

BACKGROUND

Social prescribing is the use of non-medical interventions to achieve sustained lifestyle change and improved self-care among people with long-term health conditions. As yet there is no agreed definition, although there is broad consensus that social prescribing helps patients to access non-clinical sources of support, predominantly in the community sector,[1] and is a means to address the well-documented social and economic factors that accompany long-term illness beyond the healthcare setting.[2] There is increasing interest in social prescribing as a means of addressing complex health, psychological and social issues presented in primary care, as well as its potential to reduce health inequalities.[3] A recent review of social prescribing indicates that, despite a small and largely inconclusive evidence base, there is the potential for credible psychosocial benefits to be incurred by patients with mental health problems, and for health and wellbeing improvements to be seen in people with long-term conditions.[4] To date, little attention has been paid to the impact of social prescribing on physical health and no studies have reported on the physical health status of patients.[2]

In the UK, social prescribing has been taking place on a small scale for a number of years and there are several operating models.[5] These models vary in two ways; the actual activities or services offered and the level of support given to patients following referral. Recognising that patients who are simply given information about a service will not necessarily take it up, most schemes involve a 'facilitator' coupled with personal support,[6] although the level of ongoing support offered varies considerably.

Ways to Wellness [7] is the first UK organisation to deliver social prescribing at scale. Based in West Newcastle upon Tyne, an area with some of the most socio-economically deprived wards in England, Ways to Wellness covers 18 general practices that are referring patients

For peer review only - http://bmjopen.bmj.com/site/about/guidelines.xhtml

aged 40-74 with long-term conditions (diabetes (type 1&2), chronic obstructive pulmonary disease, asthma, coronary heart disease, heart failure, epilepsy, osteoporosis) and with or without anxiety or depression to the service. Ways to Wellness is delivered by four third sector organisations and is funded for seven years through a social impact bond model, with an overall target of 11,000 users over this period.

Following extensive consultation with patients and health care professionals,[8] Ways to Wellness provides a 'hub' model of social prescribing in which a Link Worker trained in behaviour change methods offers a holistic and personalised service. Meaningful health and wellness goals are identified and service users are connected, when indicated, to community and voluntary groups and resources. The service comprises: (a) individual assessment, motivational interviewing and action planning; (b) completion of an initial 'Well-being Star' assessment and subsequent Well-being Star assessments every six months thereafter for the duration of the patient's involvement; (c) help to access community services (eg. welfare rights advice, walking groups, physical activity classes, arts groups, continuing education); (d) promotion of volunteering opportunities, and; (e) promotion of improved self-care and sustained behaviour change related to healthier lifestyle choices.

This qualitative study sought to capture the experiences of patients engaged with Ways to Wellness in its first year of operation and to identify the impact of this model of social prescribing on health and wellbeing.

For peer review only - http://bmjopen.bmj.com/site/about/guidelines.xhtml

For peer review only

METHODS

Setting

This study was set in an inner-city area of high social deprivation in the west of Newcastle upon Tyne (Population n=132000), ranked among 40th most deprived in England according to the Index of Multiple Deprivation.[9] Eighteen per cent of residents have long term conditions and receipt of sickness and disability-related benefits amongst the local population is higher than the national average.[10]

Recruitment and sampling

The four Ways to Wellness provider organisations acted as gatekeepers and approached service users on our behalf, explaining the study and issuing a participant information sheet. Those who agreed for their details to be passed on to the researcher were contacted by telephone to ascertain willingness to participate, and, if willing, an interview was arranged. We used purposive sampling across the four provider organisations based on the following criteria: age, gender; long-term condition; marital status, employment status, socio-economic status, and level of engagement with Ways to Wellness (intensive to non-intensive).

Data collection

We undertook semi-structured interviews with participants. A topic guide was developed covering: referral procedures; level and type of engagement with Ways to Wellness; goal-setting; linkage to other services; long-term condition management; changes resulting from involvement with Ways to Wellness; and, views of the service. Interviews took place in participants' homes or an alternative venue of their choosing (often where they attended their Ways to Wellness appointment). Interviews were carried out by two researchers (MS and SL). Following consent procedures, demographic details were collected. Interviews continued until data saturation was reached.[11]

For peer review only - http://bmjopen.bmj.com/site/about/guidelines.xhtml

Transcription, data management and analysis

Interviews lasted between 8 minutes and 1 hour 27 minutes (average 41 minutes), were digitally recorded and transcribed verbatim. Transcripts were anonymised and checked against recordings for accuracy. Thematic analysis was used [12] with data management supported by NVivo10 software. [13] Following close reading of the transcripts, a coding scheme was developed which contained a-priori themes based on the topic guide as well as further themes which emerged from the data. The scheme captured data relating to: referral, multi-morbidity, experiences of Ways to Wellness service delivery and onward referral, relationship with the Link Worker, impact of Ways to Wellness and barriers to service engagement. The coding framework was applied to five interviews, which were double-coded by MS and SL. The coding frame was reviewed by all team members and modifications agreed and made before being applied to all interviews. Line-by-line coding and constant comparison were used to code the entire dataset;[14 15] deviant case analysis, where we sought out opinions which modified or contradicted the analysis, was used to enhance validity.[16]

RESULTS

The impact of Ways to Wellness is described by our detailed analysis of the following three themes: negative impact of long-term conditions and multi-morbidity; Link Worker roles; and positive impact of the intervention.

Participant characteristics

As shown in Table 1, 30 adults, 14 women and 16 men, aged 40-74 (mean age 62) took part. Thirteen participants were over state pension age; four of the 17 participants of working age were in employment. Occupational social class [17] based on current or previous main employment indicates that the sample included individuals from across the social class

For peer review only - http://bmjopen.bmj.com/site/about/guidelines.xhtml

spectrum, excepting social class 1; social classes 2-4 accounted for two thirds of the sample and the remaining third were from social classes 5-8. Five participants were from Black and Minority Ethnic Communities, one identified as White Irish and the remaining 24 were White British. Multi-morbidity was a prominent feature of the sample. Based on self-reported health conditions, only one participant (P15) had a single long-term condition. Most participants had more than one Ways to Wellness long-term condition which triggered a referral, had other health problems and associated mental health issues, low confidence and social isolation.

For peer review only

For peer review only - http://bmjopen.bmj.com/site/about/guidelines.xhtml

Table 1: Demographic characteristics and long-term health conditions of study participants

ID	Sex	Employment status	Standard occupational classifications (2010) description[1, 2, 3,4]	No. of WtW conditions[5]	Total no. of non-WtW conditions[6]	Mental health / social isolation[7]	Age
1	Male	Retired	7	3	3	Yes	70
2	Female	Retired	2	3	3	Yes	73
3	Female	Employed	7	1	1	Yes	48
4	Female	Unemployed	8	2	1	Yes	58
5	Female	Retired	2	2	4	Yes	67
6	Male	Retired	4	2	1	Yes	67
7	Male	Unemployed	7	2	1	No	56
8	Female	Unemployed	2	2	3	No	59
9	Male	Employed	4	3	3	Yes	59
10	Male	Unemployed	4	2	4	Yes	64
11	Male	Unemployed	2	2	4	Yes	47
12	Male	Unemployed	4	1	3	Yes	58
13	Male	Unemployed	7	1	2	Yes	63
14	Male	Unemployed	2	2	1	No	62
15	Female	Retired	8	1	0	No	72
16	Female	Retired	2	2	0	No	67
17	Female	Unemployed	3	2	0	Yes	51
18	Female	Retired	3	3	2	Yes	65
19	Male	Retired	6	2	4	Yes	69
20	Male	Retired	4	2	1	No	70
21	Female	Retired	5	3	1	No	73
22	Female	Unemployed	3	2	2	Yes	42
23	Male	Retired	6	2	4	No	70
24	Female	Retired	2	2	0	Yes	70
25	Female	Unemployed	2	3	1	Yes	52
26	Male	Retired	3	2	3	No	73
27	Male	Employed	4	1	1	Yes	40
28	Male	Retired	4	2	1	No	74
29	Male	Employed	6	1	3	Yes	63
30	Female	Unemployed	2	2	2	Yes	48

1. SOC2010 volume 3: the National Statistics Socio-economic classification (NS-SEC rebased on SOC2010)

For peer review only - http://bmjopen.bmj.com/site/about/guidelines.xhtml

http://webarchive.nationalarchives.gov.uk/20160105160709/http://www.ons.gov.uk/ons/guide-method/classifications/current-standclassifications/soc2010/soc2010-volume-3-ns-sec--rebased-on-soc2010--user-manual/index.html . Last accessed 30 August 2016.

2. Last occupation used except when had changed due to health issues and occupation before health forced change was used for classification.

3. Occupations of NS-Sec analytical class coded using classification tool found at: https://www.ons.gov.uk/methodology/classificationsandstandards/standardoccupationalclassificationsoc/soc2010. Last accessed 21 September 2016.

4. 1 =2 = Lower managerial, administrative and professional occupation. 3 = Intermediate occupation. 4 = Small employers and own account workers. 5 = Lower supervisory and technical occupation. 6. Semi-routine occupation. 7 =Routine occupation. 8 = Never worked / long term unemployed.

5. Participants asked for details of long terms conditions.

6. Based on information given by participants and may include previous conditions.

7. A broad category that includes low mood, anxiety, depression, loneliness and social isolation and is based on self-reports in interviews where participants described or reported these conditions or feelings.

For peer review only

Impact of long-term conditions and multi-morbidity

All participants had been deeply affected, physically, emotionally and socially by their health problems. Physical effects included pain, sleep problems, side effects of medication, continence issues and significant functional limitations. With increasing age, existing conditions worsened and multi-morbidity increased:

> *I've got COPD, high blood pressure, diabetic … a prolapsed disc and … an operation on it that didn't work, so I'm in constant pain with my back. Just generally not fit, not well … stent put in because I had kidney stones. They were supposed to take it out … but because I had a temperature they didn't take it out. They left it in, but I'm having nothing but trouble with it, water infections, feeling as if I want to go to the toilet all the time and when I do go I don't pass anything. It's like dying for a wee all the time … It's got me really down.* (P1, Male, aged 70)

Some of those experiencing depression reported that this had been with them for many years, but most accounts of depression and/or anxiety were attributed to their long term condition(s) and the difficulties of carrying out a 'normal' life. Wider participation within family and community networks was affected, as well as for many, basic activities of daily living such as shopping or housework. Social isolation was common amongst many of the participants, but for those of working age, unemployment, lowered incomes and reliance on sickness welfare benefits was commonplace. The overall picture was of lives seriously blighted by long-term health problems:

I want to get back to work. I was used to doing things and it is really hard not being able to do all the things I used to do, yes, and I was depressed at the time as well … it looks like a long road to get back to being healthy again; and I needed all the help I could get … I have got asthma and I have got diabetes and high blood pressure. And since I have had the treatment for cancer… I was fine after the operation but it was the radiotherapy that really knocked me out … I mean they do warn you that it affects your energy – but my energy levels just plummeted and I wasn't able to do hardly anything. (P8, Female, aged 59)

For peer review only - http://bmjopen.bmj.com/site/about/guidelines.xhtml

Link worker roles

Connecting with service users

Link Workers operated an accessible, flexible service between 9am and 5pm, Monday-Friday. Following primary care referral, Link Workers contacted service users by telephone to arrange an initial appointment, which could be at the GP practice, community centres, cafés, or infrequently, participants' homes. Interface was more frequent in the initial stages, reducing as the relationship developed and the service user became more independent; face-face contact was also supplemented by telephone, email or text. Contact frequency decreased or increased depending on need:

> *[Link Worker] says, "I'm there, basically, any time," obviously within working hours, but she says, "Just phone me up if you need me, and any questions or anything." So basically I can see her as often as I want or as little as I want, but she likes me to keep her informed of anything happening, so she knows.* (P11, Male, aged 47)

Participants appreciated the flexibility and 'open door' nature of Ways to Wellness, although this could be limited for those who were working. Of particular value was the potential to be engaged with the service for up to two years. Due to the long term and complex nature of conditions which often fluctuated, participants recognised that a shorter term approach would be inadequate, particularly when accounting for wider life events such as job loss or bereavement:

> *Well, I think you can dip in and dip out. It's the kind of thing if you need them, you phone them and they'll get straight back to you. They're there, I know they're there … if something happens to me now* (P13, Male, aged 63)

Link worker approach

Participants consistently reported feeling at ease and relaxed with their Link Worker which enabled them to develop an open and trusting relationship. The Link Worker's person-

For peer review only - http://bmjopen.bmj.com/site/about/guidelines.xhtml

centred and non-judgemental approach facilitated trust, feelings of control and a readiness to reflect on current circumstances and implement positive changes. Service users felt listened to and valued:

> *… [Link Worker] really helped me along … they're not judgemental … and they were so easy going and they were just lovely.* (P17, Female, aged 51)
> *She [Link Worker] just brought things up … we'd have a discussion … we just started talking and that's when I just came out with different things … and she gave the feeling that you could open up to her … where some people, you are guarded because you don't want them to know too much of your private life.* (P9, Male, aged 59*)*

A central part of the Link Worker role was to facilitate engagement with other services. The level of support offered varied, and was balanced against service users' need and readiness to engage with other services. Participants appreciated not feeling under undue pressure and recounted how the right amount of encouragement supported them to engage and make changes at their own pace. Narratives indicated service users experienced a considerable degree of control over their onward referral which helped them feel that when they did engage with other services, they were ready to do so:

> *There's no pressure or anything. If you can't do it, they don't ask why, they just say, "Oh, we'll try to sort something else out for you," … She said, "Well, it needs to come from you." … if it was, like, a sort of forced thing I think I would have problems going [to self-help organisation] … I hate it if I'm made to go.* (P11, Male, aged 47*)*
> *I certainly felt better from having spoken to her [Link Worker], and for her inspiring me to … kick me along and say, "Yes, you can do that, you can do that." And when I described the [negative] feelings I had she turned them around to be something else that made me feel loads better.* (P5, Female, aged 67*)*

The level of support that some service users required in order to engage with services, particularly those involving physical activity, appeared to be considerable. Link workers paced the level of support they offered, particularly in the initial stages:

> *I just expected the Link Worker to introduce me to the gym, and that would have been it. And I think, if it had just been [that] I would have turned round, and I would have gone the opposite direction. But because of the way it was so gradually and really professionally linked in to different things, I just felt as though I'd floated into it,*

For peer review only - http://bmjopen.bmj.com/site/about/guidelines.xhtml

rather than getting shoved from behind. I just felt as though I was gradually moved into it. (P2,Female, aged 73)

Building self-confidence, self-reliance and independence was another facet of the Link Worker's approach, managed through ongoing support, persistence in finding the right motivational tools for the individual whilst conveying the need for personal responsibility and resilience. This enabled service users to make changes to their lives, engage with other organisations and manage their long-term conditions:

> *I mean, she's just someone who can make you do things… Not as in a bad way, she sort of, like, empowers you, for want of a better word, to do it, you know what I mean? She gives you, "… give it a go," and she'll explain … and if you don't go she doesn't get disappointed or anything like that, she just says, "Oh, right, well, we'll sort something else out for you." …I think the only person that can help is myself, really. I think it's 99% me and the 1% of help I get from the rest of the people, but … they can only like, give you advice and encouragement, the rest has got to be yourself.* (P11, Male, aged 47)

The holistic service offered by the Link Workers was contrasted favourably with what was available or possible through the GP or with previous attempts by other health professionals to deal with complex health issues:

> *I couldn't go to my doctors and ask them, I couldn't phone the nurse and ask the nurse.* (P13, Male, aged 63)
> *It hasn't worked miracles, but it's nice that she's there and sometimes you don't bother your GP or whatever, you know. It's a brilliant service, I think.* (P26, Male, aged 73)
> *At the very beginning, we agreed that she'd work around me, not me around her, to work on the foods that I thought were suitable for me … I met up with dieticians before … I felt they were dictating the food I should have, not the food I wanted to have. So that's what surprised me about Ways to Wellness, they're working around me … it's different. You talk about it. You're relaxed … You're in charge of it.* (P6, Male, aged 67)

For peer review only - http://bmjopen.bmj.com/site/about/guidelines.xhtml

Positive impact of the intervention

Health-related behaviours

Many participants attended physical activity classes specifically tailored to their health needs, helping them incrementally to build confidence and strength to improve their physical health. Some participants noted that as a result of the Ways to Wellness intervention, their level of fitness had improved:

> *I really enjoyed it … When I first started off, I was doing five minutes on the cycling machine and … I had that up to 20 minutes … I was up to 15 minutes on [the cross trainer] and I was pulling weights … they asked us all these relevant questions because of my medical history so I hadn't to do too much for my heart … I just feel a hell of a lot fitter.* (P28, Male, aged 74)

Participants who received healthy eating advice and cooking skills recounted in some detail how they had reduced their intake of unhealthy food and how they were equipped with the practical skills to incorporate healthier foods into their diet:

> *Certainly the* [Name of course], *because that was a six week course…The first session was salad and pizza, then we did stir fry, soup last week. Next week it's going to be fish, then I think it's desserts on the sixth week. Brilliant. It's not boring, but I just can't believe how good I've found it. It's not just for cooking. … it's what you should be eating, it's the portion size which is so important.* (P14, Male, aged 62)

> *I was asked to do a food diary. You see straightaway that what you're doing is wrong. I think that was one of the biggest things why I lost quite a bit of weight, to be honest … you don't realise at the time, but it was nothing for me to eat eight slices of bread a day. That's where all the badness is. Now, I only have pitta bread, really, or a small brown sliced bread. That was a big help, with the directions pointing to the food … it's smaller portions and more variety as well … now, I eat more fruit in the mornings and in the afternoons, and I don't pick as much.* (P29, male, aged 63)

Participants described how these diet and physical activity lifestyle changes improved their physical health. Many noted weight reduction and increased energy and fitness that enabled them to break a cycle of illness and re-start activities, such as walking, shopping and gardening, which previously they felt unable to manage:

For peer review only - http://bmjopen.bmj.com/site/about/guidelines.xhtml

At one time, when I went up [a hill] … I used to stop … but now I can walk directly up … I can carry my shopping bags now … I'd get up and dance … [without feeling] I needed to sit down because I was out of breath. (P2, Female, aged 73)

Mental health

Social isolation, low mood, anxiety and depression were commonly experienced. For some, this was related primarily to physical health, but for others, compounded by life events such as bereavement. Offering opportunities for activities which allowed people to meet and socialise in the community, reduced social isolation and positively impacted on self-confidence, self-esteem and mental wellbeing:

After [partner] passed away I was, not a recluse, but I just didn't want to talk to anybody. But since I've been coming to see [the Link Worker] I've broadened my horizons and I get out … I've got a lot more confidence. (P10, Male, aged 64)
Before the Ways to Wellness, I used to just stay in. But they explained that if you get out and about, it helps you, which has proven right … there were times when I wasn't feeling too good, and I thought, "I can't be bothered," but I've pushed myself. By the time I've got out and got back, I've felt 100% better … my health has changed a lot … it's made me feel a lot more confident that there's somebody there. (P4, Female, aged 58)

Long- term health problems were linked to a range of social and economic factors, for example, relationship breakdown, job loss, income reduction, debt and housing problems. Link Worker consultations noted socio-economic factors and, where possible, took practical action to maximise income, reduce debt and assist service users navigating the welfare benefits system:

Whatever money I owed like electricity and TV licence was in my mind always eating me from inside. I sorted out that and it just changed so many things … It changed my attitude, it changed my behaviour and it changed my mood … I am not depressed like before … I feel better about everything … I go out almost every day … I play more, I write, I do jobs at home … I read more … yes, a big change. (P30, Female, aged 48)
Because of my language barrier some things I could not sort things out so easily, but she could. Even though I didn't know about Attendance Allowance she applied for that … I didn't know which benefit I could get. She said, "I can try this one." She tried it and she was successful and it helped me a lot. (P16, Female, aged 67)

For peer review only - http://bmjopen.bmj.com/site/about/guidelines.xhtml

For those of working age, health-related unemployment was a major problem. Steps to assist with finding paid or volunteer work, returning people to work or having reasonable adjustments to work settings were undertaken and highly appreciated:

> *I felt 100% better after talking to [Link Worker], she was just excellent … She put us in contact with people [to] do a new CV and look for a new job, [and] found information out for us … which has given me the confidence to go back to my company … things were put in place at work, so that I could take my breaks when I needed them … It made a massive difference to me, personally … I was getting on board with [mental health condition], and now, I'm at work with renewed vigour.* (P9, Male, aged 59)

Long-term condition management

Weight reduction and increased fitness helped participants manage the pain and tiredness experienced as a result of their long-term health conditions. Some participants with diabetes noted being in better control of their condition due to reduced cholesterol and sugar levels. Accounts of positive improvements often followed years of worsening health and poor long-term condition management:

> *The instructor here has given me a programme that I'm working on to help build up the muscles for the legs and for my back … I'm managing [my arthritis] with the help of the gym … [also] my sugar levels … [have] come down to 4.9 … [and] my cholesterol was 3.1, which is good … all that had to do with Ways to Wellness and the exercise I've been doing.* (P6, Male, aged 67)

Long-term condition management services that service users were directed to by Link Workers were highlighted as extremely helpful, particularly the combination of expert and peer-led advice on coping and symptom management strategies:

> *The [name] group was wonderful because there were people there who had similar conditions … and they have medical professionals come in to talk about things that are specific to people with chest complaints … coping mechanisms … gentle exercises and things to help. It improves your stamina, your breathing abilities and everything. So yes, that has been absolutely wonderful … honestly it was the turning point in my life. Because I had reached rock bottom and I just needed practical help to try and get back up … you have to manage your expectations as well as your illness and it has helped considerably.* (P8, Female, aged 59)

For peer review only - http://bmjopen.bmj.com/site/about/guidelines.xhtml

Regularly engaging with services was challenging, particularly for people whose condition fluctuated and those suffering from more than one health problem. Service users worried about not always being able to attend which was often a reason for not sustaining engagement with services in the past:

> *I was really down, so I couldn't go on Friday. It's been like that since I first joined. I've been missing [sessions] because of my COPD … bad turns … chest infections … [but] they [Ways to Wellness] restarted me again, so they've looked after me … they've been really, really good … they've stuck with me.* (P1, Male, aged 70)

Participants experienced Ways to Wellness as an approach which recognised the long-term and complex nature of their health problems. It supported realistic, progressive and personalised goal-setting. Participants' expectations of progress were therefore achievable and reflected that a long-term approach was necessary to make improvements, helping people to live with, rather than cure, their conditions:

> *I am on the road but it is slow … work in progress … if it was easy I would have done it years ago … I have been well impressed [with Ways to Wellness]… because they have a very practical approach and know that it has got to be incremental … you can't do everything at once so you have got to start small and build up … they have got the big picture in mind but lots of little steps in between to get there … I would like to get back to being wonderful in the next five minutes it is not going to happen. So I know that I have got to take it bit by bit so it sticks.* (P8, Female, aged 59*)*

> *[Link Worker] epitomises the word, wellbeing, because that's what she's got in mind for you, your wellbeing. Nothing else, she gives the impression that you're the only one that she's looking after, at that time, in that moment in time, you're the only one that matters to her.* (P9, Male, aged 59)

DISCUSSION

This study provides evidence about the impact of social prescribing on health and wellbeing via improvements in health-related behaviours and long-term condition management. The specific model of social prescribing examined here comprised personalised support together with linkage into appropriate services facilitated by a Link Worker. The data reveal that key

For peer review only - http://bmjopen.bmj.com/site/about/guidelines.xhtml

elements of the model are that it is: long-term in nature; addresses the co-existence of multi-morbidity, mental health problems and social isolation; and, where applicable, tackles related socio-economic issues. This study demonstrates that the rapport and quality of the relationship between the Link Worker and service user was central to achieving wellbeing. Change in health- related behaviour and long-term condition management was facilitated through the use of setting realistic, progressive and personalised goals, problem solving, receiving regular feedback and social support. These behaviour change techniques have been shown to be effective in other lifestyle interventions. [18-20] Crucially these techniques were adopted whilst also supporting individuals to address social and economic problems. The intervention engendered feelings of control and self- confidence, reduced social isolation and led to positive physical changes such as weight loss, increased physical activity, improved long-term condition management and mental health, greater resilience and effective coping strategies to manage relapses.

Psychosocial problems, particularly mental health conditions and social isolation, are the most common reasons for referral into social prescribing interventions. [2 4] A review exploring the social prescribing evidence base identified 24 studies diverse in methodology and the service being evaluated. The quantitative evidence was limited with the exception of one randomised controlled trial demonstrating clinically important benefits in managing psychosocial needs at four months. [21] The quality of eight studies using quantitative methods was poor, with small samples and high drop-out rates.[4] Qualitative evidence, from this review and more broadly, also varies in quality, but generally indicates that those referred into social prescribing schemes report improved mental wellbeing and reduced social isolation,[4 22 23] but provide very little evidence about physical health or long-term condition management. A systematic review and meta-analysis of exercise referral schemes, a type of social prescribing intervention, targeted at sedentary individuals with or without a medical

For peer review only - http://bmjopen.bmj.com/site/about/guidelines.xhtml

diagnosis was inconclusive with regards to increasing physical activity, although the heterogeneity of the quality and the nature of the schemes is likely to have contributed to this conclusion.[24] The data presented in our study provide a much more in-depth account of *how* social prescribing positively influences long-term condition management.

Strengths, limitations and implications

The strength of this work lies in the depth and consistency of the service user accounts regarding the impact of intervention. Our sample was relatively small, but data saturation was reached [11] and we interviewed people with a wide range of long-term conditions, almost all with multi-morbidity, and many experiencing social isolation and mental health problems. Methodologically, we followed accepted practice in fieldwork, analysis and interpretation. [25] Participants were those who agreed to a referral, then enrolled into and remained with Ways to Wellness. Although we know that some study participants were initially reluctant to engage with Ways to Wellness, we cannot make claims about patients with long-term conditions who do not engage in the first place or who drop out of the programme early, an important group for further research. Our findings may be transferable [26] to other similar populations in socially disadvantaged areas were there to be a similar intervention available.

Long-term health conditions are arguably the greatest challenge facing the NHS; they have an adverse effect on quality of life and reduce life expectancy. Multi-morbidity accounts for over 70 per cent of total healthcare spend. [27] Non-medical interventions are proposed as a cost-effective alternative to foster self-management in people with long term conditions. [28,29] People from lower socio-economic groups experience higher levels of chronic disease, and also have poorer condition management, worse health outcomes, and higher mortality. [27] Behavioural risk factors for long- term conditions include poor diet, smoking and physical inactivity, all of which are socioeconomically patterned. [30] However, lifestyle behaviours are

For peer review only - http://bmjopen.bmj.com/site/about/guidelines.xhtml

also affected by wider health determinants and are not simply choices that individuals make. [31 32] Supporting people to make meaningful and sustainable lifestyle changes is complex, time consuming, and a challenge for time-limited GPs. [6] Given the complex and long-term nature of the problems faced by service users, it is unsurprising that a holistic and relatively intensive approach, as offered by Ways to Wellness, is required in order to facilitate and maintain behaviour change, and it is clearly not possible, nor appropriate to offer this level of support routinely in primary care. Referral into social prescribing programmes, is likely to continue due to the need to tackle long-term health conditions, multi-morbidity and an ageing population in the face of stretched NHS resources. [29] There is, as yet, no good quality evidence to inform the commissioning of social prescribing, [33] but there are a range of approaches to, and models of, social prescribing. [34] In order to inform practitioners and commissioners, future research should be robust and comparative, addressing when, for whom and how well the programme works, include validated measures of intervention impact and accurate costs. [33] To this we would add ethnographic research to examine potential wider impacts on families and communities and how community-based, non-medical and personalised interventions supplement health care.

For peer review only - http://bmjopen.bmj.com/site/about/guidelines.xhtml

Acknowledgements

We thank the participants for sharing their experiences, Link Workers and Link Worker Managers at Mental Health Concern, First Contact Clinical, Healthworks and Changing Lives for their assistance with recruitment, and Chris Drinkwater, Tara Case and Amie Callan from Ways to Wellness for their support.

Contributors

SM, LP and NO'B conceived and designed the research and obtained funding. MS and SL undertook interviews. SM, MS, LP and SL and contributed to the data analysis. SM, MS, SL, LP and NO'B wrote the paper. All authors read and approved the final version.

Funding

This work was funded by the Cabinet Office of the UK Government (grant number A1543). The researchers are independent of the funders and the views and opinions expressed in this paper are those of the authors and not necessarily those of the Cabinet Office. The funders and sponsors (Newcastle University) had no role in the design, collection, analysis and interpretation of the data, or the writing of this paper or the decision to submit this article for publications. All authors had full access to the data in the study and can take full responsibility for the integrity of the data and the accuracy of the analysis.

Competing interests

None

For peer review only - http://bmjopen.bmj.com/site/about/guidelines.xhtml

Ethics approval

This study was approved by Newcastle University Faculty of Medical Sciences Research Ethics Committee (00868/2014)

Data sharing statement

No additional data are available

For peer review only

For peer review only - http://bmjopen.bmj.com/site/about/guidelines.xhtml

References

1. South J, Higgins TJ. Can social prescribing provide the missing link? *Primary Health Care Research and Development* 2008;9:310 - 18.

2. Mossabir R, Morris R, Kennedy A, et al. A scoping review to understand the effectiveness of linking schemes from healthcare providers to community resources to improve the health and well-being of people with long-term conditions. *Health & Social Care in the Community* 2015;23(5):467-84. doi: 10.1111/hsc.12176

3. Popay J, Kowarzik U, Mallinson S, et al. Social problems, primary care and pathways to help and support: addressing health inequalities at the individual level. part I: the GP perspective. *Journal of Epidemiology and Community Health* 2007;61(11):966-71. doi: 10.1136/jech.2007.061937

4. Kilgarriff-Foster A, O'Cathain A. Exploring the components and impact of social prescribing. *Journal of Public Mental Health* 2015;14(3):127-34.

5. Husk K, Blockley K, Lovell R, et al. What approaches to social prescribing work, for whom, and in what circumstances? A protocol for a realist review. *Systematic reviews* 2016;5(1):93-93. doi: 10.1186/s13643-016-0269-6

6. Brandling J, House W. Social prescribing in general practice: adding meaning to medicine. *British Journal of General Practice* 2009;59(563):454-56. doi: 10.3399/bjgp09X421085

7. Ways to Wellness http://waystowellness.org.uk/ 2016 (accessed 16 May 2016)

8. NHS North East Innovation Fund. Year of Care, Thanks for the Petunias- a guide to developing and commissioning non-traditional providers to support the self management of people with long term conditions, 2011.

For peer review only - http://bmjopen.bmj.com/site/about/guidelines.xhtml

9. Department for Communities and Local Government. The English Indices of Deprivation 2010, 2013, https://www.gov.uk/government/statistics/english-indices-of-deprivation-2010 (accessed 4 Aug 2016)

10. Newcastle West Clinical Commissioning Group. Annual Report and Accounts 2013-14. Newcastle, 2014.

11. Denzin NK, Lincoln YS. Handbook of qualitative research. London: Sage 2000.

12. Green J, Thorogood N. Qualitative Methods for Health Research. London: Sage 2014.

13. NVivo 10. Computer Assisted Software for Qualitative Analysis 2010 [accessed 20.3.07 2007.

14. Glaser B, Strauss A. The Discovery of Grounded Theory. Chicago: Aldine 1967.

15. Silverman D. Doing qualitative research. London: Sage 2000.

16. Barbour RS. Checklists for improving rigour in qualitative research: a case of the tail wagging the dog? *BMJ : British Medical Journal* 2001;322(7294):1115-17.

17. ONS. SOC2010 volume 3: the National Statistics Socio-economic classification (NS-SEC rebased on SOC2010) 2010 [Available from: http://webarchive.nationalarchives.gov.uk/20160105160709/http://www.ons.gov.uk/ons/guide-method/classifications/current-standard-classifications/soc2010/soc2010-volume-3-ns-sec--rebased-on-soc2010--user-manual/index.html (accessed 5 Oct 2016).

18. Greaves C, Sheppard K, Abraham C, et al. Systematic review of reviews of intervention components associated with increased effectiveness in dietary and physical activity interventions. *BMC Public Health* 2011;11(1):119.

19. Lara J, Evans EH, O'Brien N, et al. Association of behaviour change techniques with effectiveness of dietary interventions among adults of retirement age: a systematic

For peer review only

For peer review only - http://bmjopen.bmj.com/site/about/guidelines.xhtml

review and meta-analysis of randomised controlled trials. *BMC Medicine* 2014;12 doi: 10.1186/s12916-014-0177-3

20. O'Brien N, McDonald S, Araujo-Soares V, et al. The features of interventions associated with long-term effectiveness of physical activity interventions in adults aged 55-70 years: a systematic review and meta-analysis. *Health Psychology Review* 2015;9(4):417-33. doi: 10.1080/17437199.2015.1012177
21. Grant C, Goodenough T, Harvey I, et al. A randomised controlled trial and economic evaluation of a referrals facilitator between primary care and the voluntary sector. *BMJ* 2000;320:419-23.
22. Kimberlee R, Ward R, Jones M, et al. Proving our values: Measuring the economic impact of Well Spring Healthy LIving Centre's Social Prescribing for low level mental health isues encountered by GP services. Bristol: University of the West of England, 2014.
23. City and Hackney Clinical Commissioning Group and University of East London. Shine 2014 final report: Social prescribing: integrating GP and community assets for health, 2015.
24. Pavay TG, Taylor AH, Fox KR, et al. Effect of exercise referral schemes in primary care on physical activity and improving health outcomes: systematic review and meta-analysis. *BMJ* 2011;343:d6462.
25. Barbour R, S. The Newfound Credibility of Qualitative Research? Tales of Technical Essentialism and Co-Option. *Qualitative Health Research* 2003;13(7):1019-27.
26. Ritchie J, Lewis J. Qualitative Research Practice. A Guide for Social Scientists. London: Sage 2003.

For peer review only - http://bmjopen.bmj.com/site/about/guidelines.xhtml

27. Barnett K, Mercer SW, Norbury M, et al. Epidemiology of multimorbidity and implications for health care, research, and medical education: a cross-sectional study. *The Lancet* 2012;380(9836):37-43.

28. Department of Health. Our health, Our Care, Our Say: A New Direction for Community Services. 2006. http://www.dh.gov.uk/en/Publicationsandstatistics/Publications/PublicationsPolicyAndGuidance/DH_4127453 (accessed March 2015)

29. Coulter A, Roberts S, A. D. Deliversing better servgices for people with long-term contitions: building the house of care. London: The King's Fund, 2013. https://www.kingsfund.org.uk/sites/files/kf/field/field_publication_file/delivering-better-services-for-people-with-long-term-conditions.pdf (accessed 14 Nov 2016)

30. Wardle J, Steptoe A. Socioeconomic differences in attitudes and beliefs about healthy lifestyles. *Journal of Epidemiology and Community Health* 2003;57(6):440-43. doi: 10.1136/jech.57.6.440

31. UCL Institute of Health Equity. Tackling Health Inequalities Through Action on the Social Determinants of Health http://www.instituteofhealthequity.org/projects/tackling-health-inequalities-through-action-on-the-social-determinants-of-health-lessons-from-experience 2014 (accessed 8 Jan 2015_

32. Salmon P, Hall GM. Patient empowerment and control: a psychological discourse in the service of medicine. *Social Science & Medicine,* 2003;57:1969-80.

33. Centre for Reviews and Dissemination. Evidence to inform the commissioning of social prescribing. York: The University of York, https://www.york.ac.uk/media/crd/Ev%20briefing_social_prescribing.pdf, 2015. (accessed 4 Aug 2016)

For peer review only - http://bmjopen.bmj.com/site/about/guidelines.xhtml

34. Kimberlee R. What is social prescribing. *Advances in Social Sciences Research Journal* 2015;2(102-110)

For peer review only

For peer review only - http://bmjopen.bmj.com/site/about/guidelines.xhtml

BMJ Open

Link worker social prescribing to improve health and wellbeing for people with long-term conditions: qualitative study of service user perceptions

Journal:	*BMJ Open*
Manuscript ID	bmjopen-2016-015203.R1
Article Type:	Research
Date Submitted by the Author:	17-Jan-2017
Complete List of Authors:	Moffatt, Suzanne; Newcastle University, Institute of Health and Society<br>Steer, Mel; Newcastle University, Institute of Health and Society<br>Lawson, Sarah; Newcastle University, Institute of Health and Society<br>Penn, Linda; Newcastle University, Institute of Health and Society<br>O'Brien, Nicola; Newcastle University, Institute of Health and Society
**Primary Subject Heading**:	Public health
Secondary Subject Heading:	Qualitative research
Keywords:	Social prescribing, Long-term condition management, Health inequalities, Multi-morbidity

SCHOLARONE™
Manuscripts

For peer review only

Link Worker social prescribing to improve health and wellbeing for people with long-term conditions: qualitative study of service user perceptions.

Suzanne Moffatt, PhD[1]

M Steer, PhD

S Lawson, BA (Hons)

L Penn, PhD

N O'Brien, PhD

Word count: 4067 (excluding table, quotes and references)

Word count: 6139 (including table and quotes, excluding references)

1.Corresponding author

Suzanne Moffatt

Senior Lecturer in Social Gerontology

Institute of Health and Society

Newcastle University

Campus for Ageing and Vitality

Biomedical Research Building

Newcastle upon Tyne, NE4 5PL

Email: suzanne.moffatt@ncl.ac.uk

Tel +44 (0) 191 208 5005

For peer review only

For peer review only - http://bmjopen.bmj.com/site/about/guidelines.xhtml

ABSTRACT

Objectives: To describe the experiences of patients with long-term conditions who are referred to and engage with a Link Worker social prescribing intervention and identify the impact of the intervention on their health and wellbeing.

Design: Qualitative study using semi-structured interviews with thematic analysis of the data.

Intervention: Link Worker social prescribing comprising personalised support to identify meaningful health and wellness goals, ongoing support to achieve agreed objectives and linkage into appropriate community services.

Setting: Inner-city area in West Newcastle upon Tyne, UK (population N=132,000) ranked 40th most socio-economically deprived in England, served by 17 general practices.

Participants: Thirty adults with long-term conditions, 14 female, 16 male aged 40-74, mean age 62 years, 24 White British, 1 White Irish, five from Black and Minority Ethnic communities.

Results: Most participants experienced multi-morbidity combined with mental health problems, low self-confidence and social isolation. All were adversely affected physically, emotionally and socially by their health problems. The intervention engendered feelings of control and self-confidence, reduced social isolation and had a positive impact on health-related behaviours including weight loss, healthier eating and increased physical activity. Management of long-term conditions and mental health in the face of multi-morbidity improved and participants reported greater resilience and more effective problem solving strategies.

Conclusions: Findings suggest that tackling complex and long-term health problems requires an extensive holistic approach not possible in routine primary care. This model of social

For peer review only

prescribing, which takes into account physical, mental health, social and economic issues, was successful for patients who engaged with the service. Future research on a larger scale is required to assess when and for whom social prescribing is clinically and cost effective.

For peer review only

For peer review only - http://bmjopen.bmj.com/site/about/guidelines.xhtml

Strengths and limitations of this study

- This is the first UK study to provide detailed insight into the impact of Link Worker social prescribing on health and wellbeing among adults with long-term conditions living in an area of high socio-economic deprivation.
- Our interview data showed that the social prescribing intervention engendered feelings of control and self-confidence, reduced social isolation and led to positive physical and behavioural changes such as weight loss, increased physical activity, improved mental health and long-term condition management as well as greater resilience and effective coping strategies to manage relapses.
- Key elements of the social prescribing model are that it: addresses the co-existence of multi-morbidity, mental health problems and social isolation; is long-term in nature; and, where applicable, tackles related socio-economic issues.
- A strength of this work was the depth and consistency of participant accounts regarding the impact of the intervention. A limitation was that we did not capture the experiences of patients with long-term conditions who refuse a referral or who drop out of the programme early.
- Our study adds to the emerging evidence base on Link Worker social prescribing by demonstrating improvements in health-related behaviours and long-term condition management.

For peer review only - http://bmjopen.bmj.com/site/about/guidelines.xhtml

BACKGROUND

Social prescribing enables health care practitioners to refer patients to a range of non-clinical services. [1] Primarily, but not solely, directed at people with long term conditions, social prescribing harnesses assets within the voluntary and community sector to improve and encourage self-care and facilitate health-creating communities. [2 3 4] There is increasing interest in social prescribing as a means of addressing complex health, psychological and social issues presented in primary care, as well as its potential to reduce health inequalities.[5] A recent review of social prescribing indicates that, despite a small and largely inconclusive evidence base, there is the potential for credible psychosocial benefits to be incurred by patients with mental health problems, and for health and wellbeing improvements to be seen in people with long-term conditions.[6] Whilst less attention has been paid to the impact of social prescribing on physical health and resource use, [4] improvements in physical activity, [7] reductions in hospital resource use, [8] and GP attendance [9] have been attributed to social prescribing, although longer-term studies with larger sample sizes are required for more definitive evidence. [4]

As yet there is no agreed definition of social prescribing, [2] although there is broad consensus that it helps patients to access non-clinical sources of support, predominantly in the community sector,[3] and is a means to address the well-documented social and economic factors that accompany long-term illness beyond the healthcare setting.[4] In the UK, social prescribing has been taking place on a small scale for a number of years and there are several operating models.[10] These models vary in two ways; the actual activities or services offered and the level of support given to patients following referral. Recognising that patients who are simply given information about a service will not necessarily take it up, most schemes involve a ‘facilitator’ coupled with personal support,[11] although the level of ongoing support offered varies considerably. Services into which patients are referred vary, and can include

For peer review only - http://bmjopen.bmj.com/site/about/guidelines.xhtml

activities that involve physical activity such as gyms, walking groups, gardening or dance clubs; weight management and healthy eating activities, such as cooking clubs. Addressing wider economic and social issues can involve referral into services which address welfare, debt, housing and employment issues. Groups, such as those targeted at people with specific long term conditions, for example diabetes, chronic obstructive pulmonary disease, may also be accessed via social prescription. Our definition concurs with that of the social prescribing network of Ireland and Great Britain, "enabling healthcare professionals to refer patients to a link worker, to co-design a non-clinical social prescription to improve their health and wellbeing" and use services provided by the voluntary and community sector. (p19) [2]

Ways to Wellness [12] is one of the first UK organisations to deliver social prescribing on a large and prolonged scale; funded for seven years through a social impact bond model, with an overall target of 11,000 users over this period. Based in West Newcastle upon Tyne, an area with some of the most socio-economically deprived wards in England, Ways to Wellness covers 17 general practices that are referring patients aged 40-74 with long-term conditions (diabetes (type 1&2)), chronic obstructive pulmonary disease, asthma, coronary heart disease, heart failure, epilepsy, osteoporosis) and with or without anxiety or depression to the service. Ways to Wellness is delivered by four voluntary sector organisations.

Following extensive consultation with patients and health care professionals over an eight year period,[13] Ways to Wellness provides a 'hub' model of social prescribing in which a Link Worker trained in behaviour change methods offers a holistic and personalised service. Following referral from a primary care practitioner (GP, practice nurse, health care assistant), meaningful health and wellness goals are jointly identified and service users are connected, when desired, to community and voluntary groups and resources. The service comprises: (a)

For peer review only - http://bmjopen.bmj.com/site/about/guidelines.xhtml

individual assessment, motivational interviewing and action planning; (b) completion of an initial ‘Well-being Star’ assessment and subsequent Well-being Star assessments every six months thereafter for the duration of the patient’s involvement; (c) help to access community services (e.g. welfare rights advice, walking groups, physical activity classes, arts groups, continuing education); (d) promotion of volunteering opportunities, and; (e) promotion of improved self-care and sustained behaviour change related to healthier lifestyle choices. Thus, the intervention is highly individualised with patient engagement varying in terms of intensity, duration, personalised goal-setting and onward referral. Patients can remain with the intervention for up to two years, but with Link Worker discretion beyond two years if required; frequency of contact with the Link Worker is mutually agreed, varies between and within patients depending on current need and circumstances, and can be face to face, via telephone, email and/or text message. Data for this study were collected in the first 14 months of intervention implementation. To date, the four provider organisations have ‘linked’ patients into 133 different community and voluntary sector organisations which can be grouped as follows: long-term condition management; mental health; physical activity; weight management and healthy eating; addiction; welfare rights advice; emergency financial assistance; debt; housing; volunteering/learning/employment; and community activities including gardening, cooking, art, crafts.

This qualitative study sought to capture the experiences of patients engaged with Ways to Wellness in its 14 months of operation and to identify the impact of this model of Link Worker social prescribing on health and wellbeing.

For peer review only

For peer review only - http://bmjopen.bmj.com/site/about/guidelines.xhtml

METHODS

Setting

This study was set in an inner-city area of high socio-economic deprivation in the west of Newcastle upon Tyne (population n=132000), ranked among 40th most deprived in England according to the Index of Multiple Deprivation.[14] Eighteen per cent of residents have long-term conditions and receive sickness and disability-related benefits, which is higher than the national average.[15]

Recruitment and sampling

The four Ways to Wellness provider organisations acted as gatekeepers and approached service users on our behalf, explaining the study and issuing a participant information sheet. Those who agreed for their details to be passed on to the researcher were contacted by telephone to ascertain willingness to participate, and, if willing, an interview was arranged. We used purposive sampling across the four provider organisations based on the following criteria: age, gender; long-term condition; marital status, employment status, socio-economic status, and level of engagement with Ways to Wellness (intensive to non-intensive). For the purposes of sampling, long-term condition was ascertained by the Link Worker via the referral form, and had therefore been diagnosed by a GP or other health care professional.

Data collection

We undertook one semi-structured interview with each participant between January and June 2016 whilst they were engaged with Ways to Wellness (length of engagement ranged from 4-14 months). A topic guide was developed covering: referral procedures; level and type of engagement with Ways to Wellness; goal-setting; linkage to other services; long-term condition management; changes resulting from involvement with Ways to Wellness; and views of the service. Interviews took place in participants' homes or an alternative venue of

For peer review only - http://bmjopen.bmj.com/site/about/guidelines.xhtml

their choosing (often where they attended their Ways to Wellness appointment). Interviews were carried out by two researchers (MS and SL). Following consent procedures, demographic details were collected. Interviews continued until data saturation was reached.[16]

Transcription, data management and analysis

Interviews lasted between eight minutes and one hour 27 minutes (average 41 minutes), were digitally recorded and transcribed verbatim. Fieldnotes were made immediately after each interview and shared amongst the team. Transcripts were anonymised and checked against recordings for accuracy. Thematic analysis was used [17] with data management supported by NVivo10 software. [18] Following close reading of the transcripts, a coding scheme was developed which contained a-priori themes based on the topic guide as well as further themes which emerged from the data. The scheme captured data relating to: referral, multi-morbidity, experiences of Ways to Wellness service delivery and onward referral, relationship with the Link Worker, impact of Ways to Wellness and barriers to service engagement. The coding framework was applied to an initial randomly selected five interviews, which were double-coded by MS and SL. Following this, the coding frame was reviewed by all team members, modifications agreed and made before being applied to all interviews. Line-by-line coding and constant comparison were used to code the entire dataset;[19 20] deviant case analysis, where we sought out opinions which modified or contradicted the analysis, was used to enhance validity.[21]

RESULTS

The impact of Ways to Wellness is described by our detailed analysis of the following three themes: negative impact of long-term conditions and multi-morbidity; Link Worker roles; and positive impact of the intervention.

Participant characteristics

For peer review only - http://bmjopen.bmj.com/site/about/guidelines.xhtml

As shown in Table 1, 30 adults, 14 women and 16 men, aged 40-74 (mean age 62) took part. Thirteen participants were over state pension age; four of the 17 participants of working age were in employment. Occupational social class [22] based on current or previous main employment indicates that the sample included individuals from across the social class spectrum, excepting social class 1; social classes 2-4 accounted for two thirds of the sample and the remaining third were from social classes 5-8. Five participants were from Black and Minority Ethnic Communities, one identified as White Irish and the remaining 24 were White British. Multi-morbidity was a prominent feature of the sample. Based on self-reported health conditions, only one participant (P15) had a single long-term condition. Most participants had more than one long-term condition which triggered a referral, had other health problems and associated mental health issues, low confidence and social isolation. With the exception of the diagnosed long-term conditions that triggered a referral to Ways to Wellness (i.e. diabetes (type 1&2), chronic obstructive pulmonary disease, asthma, coronary heart disease, heart failure, epilepsy, osteoporosis) other physical and mental health problems were self-reported at interview. No further assessment of physical or mental health was made during the study.

At the time of interview, participants had been receiving the intervention for between four and fourteen months. Services that participants reported being linked into were as follows: physical activity/fitness, weight management, healthy eating, long term condition management (i.e. support groups specifically aimed at assisting people manage their long term conditions), welfare rights services (including managing debt, applying for welfare benefits and/or emergency hardship funds), housing advice, employment advice services, community activities (e.g. choir, swimming, fishing, photography) and other voluntary organisations (e.g. Age UK).

For peer review only - http://bmjopen.bmj.com/site/about/guidelines.xhtml

Table 1: Demographic characteristics and long-term health conditions of study participants

ID	Sex	Employment status	Occupational social class [1, 2, 3,4]	No. of WtW conditions[5]	Total no. of non-WtW conditions[6]	Mental health / social isolation[7]	Age band	Months involved with WtW at interview
1	Male	Retired	7	3	3	Yes	70-74	4
2	Female	Retired	2	3	3	Yes	70-74	4
3	Female	Employed	7	1	1	Yes	45-49	5
4	Female	Unemployed	8	2	1	Yes	55-59	5
5	Female	Retired	2	2	4	Yes	65-69	6
6	Male	Retired	4	2	1	Yes	65-69	7
7	Male	Unemployed	7	2	1	No	55-59	7
8	Female	Unemployed	2	2	3	No	55-59	7
9	Male	Employed	4	3	3	Yes	55-59	7
10	Male	Unemployed	4	2	4	Yes	60-64	7
11	Male	Unemployed	2	2	4	Yes	45-49	7
12	Male	Unemployed	4	1	3	Yes	55-59	7
13	Male	Unemployed	7	1	2	Yes	60-64	7
14	Male	Unemployed	2	2	1	No	60-64	7
15	Female	Retired	8	1	0	No	70-74	8
16	Female	Retired	2	2	0	No	65-69	8
17	Female	Unemployed	3	2	0	Yes	50-54	9
18	Female	Retired	3	3	2	Yes	65-69	10
19	Male	Retired	6	2	4	Yes	65-69	10
20	Male	Retired	4	2	1	No	70-74	11
21	Female	Retired	5	3	1	No	70-74	12
22	Female	Unemployed	3	2	2	Yes	40-44	12
23	Male	Retired	6	2	4	No	70-74	12
24	Female	Retired	2	2	0	Yes	70-74	12
25	Female	Unemployed	2	3	1	Yes	50-54	12
26	Male	Retired	3	2	3	No	70-74	13
27	Male	Employed	4	1	1	Yes	40-44	14
28	Male	Retired	4	2	1	No	70-74	14
29	Male	Employed	6	1	3	Yes	60-64	NK[8]
30	Female	Unemployed	2	2	2	Yes	45-49	NK[8]

For peer review only - http://bmjopen.bmj.com/site/about/guidelines.xhtml

1. SOC2010 volume 3: the National Statistics Socio-economic classification (NS-SEC rebased on SOC2010) http://webarchive.nationalarchives.gov.uk/20160105160709/http://www.ons.gov.uk/ons/guide-method/classifications/current-standclassifications/soc2010/soc2010-volume-3-ns-sec--rebased-on-soc2010--user-manual/index.html . Last accessed 30 August 2016.

2. Last occupation used except when had changed due to health issues and occupation before health forced change was used for classification.

3. Occupations of NS-Sec analytical class coded using classification tool found at: https://www.ons.gov.uk/methodology/classificationsandstandards/standardoccupationalclassificationsoc/soc2010. Last accessed 21 September 2016.

4. 1 =2 = Lower managerial, administrative and professional occupation. 3 = Intermediate occupation. 4 = Small employers and own account workers. 5 = Lower supervisory and technical occupation. 6. Semi-routine occupation. 7 =Routine occupation. 8 = Never worked / long term unemployed.

5. Conditions which triggered a referral to Ways to Wellness were: diabetes (type 1&2), chronic obstructive pulmonary disease, asthma, coronary heart disease, heart failure, epilepsy, osteoporosis) with or without anxiety or depression. This column indicates the number of 'Ways to Wellness' long-term conditions that participants had been diagnosed with by a medical practitioner.

6. Based on self-report at interview.

7. A broad category that includes low mood, anxiety, depression, loneliness and social isolation and is based on self-report at interview where participants described or reported these conditions or feelings.

8. Not known

For peer review only - http://bmjopen.bmj.com/site/about/guidelines.xhtml

Impact of long-term conditions and multi-morbidity

All participants had been deeply affected, physically, emotionally and socially by their health problems. Physical effects included pain, sleep problems, side effects of medication, continence issues and significant functional limitations. With increasing age, existing conditions worsened and multi-morbidity increased:

> *I've got COPD, high blood pressure, diabetic … a prolapsed disc and … an operation on it that didn't work, so I'm in constant pain with my back. Just generally not fit, not well … stent put in because I had kidney stones. They were supposed to take it out … but because I had a temperature they didn't take it out. They left it in, but I'm having nothing but trouble with it, water infections, feeling as if I want to go to the toilet all the time and when I do go I don't pass anything. It's like dying for a wee all the time … It's got me really down.* (P1, Male, aged 70)

Some of those experiencing depression reported that this had been with them for many years, but most accounts of depression and/or anxiety were attributed to their long-term condition(s) and the difficulties of carrying out a 'normal' life. Wider participation within family and community networks was affected, as well as basic activities of daily living such as shopping or housework. Social isolation was common amongst many of the participants, but for those of working age, unemployment, lowered incomes and reliance on sickness welfare benefits was commonplace. The overall picture was of lives seriously blighted by long-term health problems:

I want to get back to work. I was used to doing things and it is really hard not being able to do all the things I used to do, yes, and I was depressed at the time as well … it looks like a long road to get back to being healthy again; and I needed all the help I could get … I have got asthma and I have got diabetes and high blood pressure. And since I have had the treatment for cancer… I was fine after the operation but it was the radiotherapy that really knocked me out … I mean they do warn you that it affects your energy – but my energy levels just plummeted and I wasn't able to do hardly anything. (P8, Female, aged 59)

For peer review only - http://bmjopen.bmj.com/site/about/guidelines.xhtml

Link Worker roles

Connecting with service users

Link Workers operated an accessible, flexible service between 9am and 5pm, Monday-Friday. Following primary care referral, Link Workers contacted service users by telephone to arrange an initial appointment, which could be held at GP practices, community centres, cafés, or infrequently, participants' homes. In general, intervention contacts were more frequent at the initial stages, reducing as the relationship developed and the service user became more independent; however, contact also decreased or increased with current need. Face-to-face contact was also supplemented by telephone, email or text:

> *[Link Worker] says, "I'm there, basically, any time," obviously within working hours, but she says, "Just phone me up if you need me, and any questions or anything." So basically I can see her as often as I want or as little as I want, but she likes me to keep her informed of anything happening, so she knows.* (P11, Male, aged 47)

Participants appreciated the flexibility and 'open door' nature of Ways to Wellness, although this could be limited for those who were working. Of particular value was the potential to be engaged with the service for up to two years. Due to the long-term and complex nature of conditions which often fluctuated, participants recognised that a shorter term approach would be inadequate, particularly when accounting for wider life events such as job loss or bereavement:

> *Well, I think you can dip in and dip out. It's the kind of thing if you need them, you phone them and they'll get straight back to you. They're there, I know they're there … if something happens to me now* (P13, Male, aged 63)

Link Worker approach

Participants consistently reported feeling at ease and relaxed with their Link Worker which enabled them to develop an open and trusting relationship. The Link Worker's person-

For peer review only - http://bmjopen.bmj.com/site/about/guidelines.xhtml

centred and non-judgemental approach facilitated trust, feelings of control and a readiness to reflect on current circumstances and implement positive changes. Service users felt listened to and valued:

> *... [Link Worker] really helped me along ... they're not judgemental ... and they were so easy going and they were just lovely.* (P17, Female, aged 51)
> *She [Link Worker] just brought things up ... we'd have a discussion ... we just started talking and that's when I just came out with different things ... and she gave the feeling that you could open up to her ... where some people, you are guarded because you don't want them to know too much of your private life.* (P9, Male, aged 59*)*

A central part of the Link Worker role was to facilitate engagement with other services. The level of support offered varied, and was balanced against service users' need and readiness to engage with other services. Participants appreciated not feeling under undue pressure and recounted how the right amount of encouragement supported them to engage and make changes at their own pace. Narratives indicated service users experienced a considerable degree of control over their onward referral which helped them feel that when they did engage with other services, they were ready to do so:

> *There's no pressure or anything. If you can't do it, they don't ask why, they just say, "Oh, we'll try to sort something else out for you," ... She said, "Well, it needs to come from you." ... if it was, like, a sort of forced thing I think I would have problems going [to self-help organisation] ... I hate it if I'm made to go.* (P11, Male, aged 47*)*
> *I certainly felt better from having spoken to her [Link Worker], and for her inspiring me to ... kick me along and say, "Yes, you can do that, you can do that." And when I described the [negative] feelings I had she turned them around to be something else that made me feel loads better.* (P5, Female, aged 67*)*

The level of support that some service users required in order to engage with services, particularly those involving physical activity, appeared to be considerable. Link workers paced the level of support they offered, particularly in the initial stages:

> *I just expected the Link Worker to introduce me to the gym, and that would have been it. And I think, if it had just been [that] I would have turned round, and I would have gone the opposite direction. But because of the way it was so gradually and really professionally linked in to different things, I just felt as though I'd floated into it,*

For peer review only - http://bmjopen.bmj.com/site/about/guidelines.xhtml

rather than getting shoved from behind. I just felt as though I was gradually moved into it. (P2,Female, aged 73)

Building self-confidence, self-reliance and independence was another facet of the Link Worker's approach, managed through ongoing support and persistence in finding the right motivational tools for the individual, whilst conveying the need for personal responsibility and resilience. This enabled service users to make changes to their lives, engage with other organisations and manage their long-term conditions:

I mean, she's just someone who can make you do things… Not as in a bad way, she sort of, like, empowers you, for want of a better word, to do it, you know what I mean? She gives you, "… give it a go," and she'll explain … and if you don't go she doesn't get disappointed or anything like that, she just says, "Oh, right, well, we'll sort something else out for you." …I think the only person that can help is myself, really. I think it's 99% me and the 1% of help I get from the rest of the people, but … they can only like, give you advice and encouragement, the rest has got to be yourself. (P11, Male, aged 47)

The holistic service offered by the Link Workers was contrasted favourably with what was available or possible through the GP or with previous attempts by other health professionals to deal with complex health issues:

I couldn't go to my doctors and ask them, I couldn't phone the nurse and ask the nurse. (P13, Male, aged 63)
It hasn't worked miracles, but it's nice that she's there and sometimes you don't bother your GP or whatever, you know. It's a brilliant service, I think. (P26, Male, aged 73)
At the very beginning, we agreed that she'd work around me, not me around her, to work on the foods that I thought were suitable for me … I met up with dieticians before … I felt they were dictating the food I should have, not the food I wanted to have. So that's what surprised me about Ways to Wellness, they're working around me … it's different. You talk about it. You're relaxed … You're in charge of it. (P6, Male, aged 67)

For peer review only - http://bmjopen.bmj.com/site/about/guidelines.xhtml

Positive impact of the intervention

Health-related behaviours

Many participants attended physical activity classes specifically tailored to their health needs, helping them incrementally to build confidence and strength to improve their physical health. Some participants noted that as a result of the Ways to Wellness intervention, their level of fitness had improved:

> *I really enjoyed it … When I first started off, I was doing five minutes on the cycling machine and … I had that up to 20 minutes … I was up to 15 minutes on [the cross trainer] and I was pulling weights … they asked us all these relevant questions because of my medical history so I hadn't to do too much for my heart … I just feel a hell of a lot fitter.* (P28, Male, aged 74)

Participants who received healthy eating advice and cooking skills recounted in some detail how they had reduced their intake of unhealthy food and how they were equipped with the practical skills to incorporate healthier foods into their diet:

> *Certainly the* [Name of course], *because that was a six week course…The first session was salad and pizza, then we did stir fry, soup last week. Next week it's going to be fish, then I think it's desserts on the sixth week. Brilliant. It's not boring, but I just can't believe how good I've found it. It's not just for cooking. … it's what you should be eating, it's the portion size which is so important.* (P14, Male, aged 62)

> *I was asked to do a food diary. You see straightaway that what you're doing is wrong. I think that was one of the biggest things why I lost quite a bit of weight, to be honest … you don't realise at the time, but it was nothing for me to eat eight slices of bread a day. That's where all the badness is. Now, I only have pitta bread, really, or a small brown sliced bread. That was a big help, with the directions pointing to the food … it's smaller portions and more variety as well … now, I eat more fruit in the mornings and in the afternoons, and I don't pick as much.* (P29, male, aged 63)

Participants described how these diet and physical activity lifestyle changes improved their physical health. Many noted weight reduction and increased energy and fitness that enabled them to break a cycle of illness and re-start activities, such as walking, shopping and gardening, which previously they felt unable to manage:

For peer review only - http://bmjopen.bmj.com/site/about/guidelines.xhtml

At one time, when I went up [a hill]… I used to stop … but now I can walk directly up … I can carry my shopping bags now … I'd get up and dance … [without feeling] I needed to sit down because I was out of breath. (P2, Female, aged 73)

Mental health

Social isolation, low mood, anxiety and depression were commonly experienced. For some, this was related primarily to physical health, but for others, this was compounded by life events such as bereavement. Offering opportunities for activities, which allowed people to meet and socialise in the community, reduced social isolation and positively impacted on self-confidence, self-esteem and mental wellbeing:

After [partner] passed away I was, not a recluse, but I just didn't want to talk to anybody. But since I've been coming to see [the Link Worker] I've broadened my horizons and I get out … I've got a lot more confidence. (P10, Male, aged 64)
Before the Ways to Wellness, I used to just stay in. But they explained that if you get out and about, it helps you, which has proven right … there were times when I wasn't feeling too good, and I thought, "I can't be bothered," but I've pushed myself. By the time I've got out and got back, I've felt 100% better … my health has changed a lot … it's made me feel a lot more confident that there's somebody there. (P4, Female, aged 58)

Long-term health problems were linked to a range of social and economic factors. including relationship breakdown, job loss, income reduction, debt and housing problems. Link Worker consultations noted socio-economic factors and, where possible, took practical action to maximise income, reduce debt and assist service users navigating the welfare benefits system:

Whatever money I owed like electricity and TV licence was in my mind always eating me from inside. I sorted out that and it just changed so many things … It changed my attitude, it changed my behaviour and it changed my mood … I am not depressed like before … I feel better about everything … I go out almost every day … I play more, I write, I do jobs at home … I read more … yes, a big change. (P30, Female, aged 48)
Because of my language barrier some things I could not sort things out so easily, but she could. Even though I didn't know about Attendance Allowance she applied for that … I didn't know which benefit I could get. She said, "I can try this one." She tried it and she was successful and it helped me a lot. (P16, Female, aged 67)

For peer review only - http://bmjopen.bmj.com/site/about/guidelines.xhtml

For those of working age, health-related unemployment was a major problem. Steps to assist with finding paid or volunteer work, returning people to work or having reasonable adjustments to work settings were undertaken and highly appreciated:

> *I felt 100% better after talking to [Link Worker], she was just excellent … She put us in contact with people [to] do a new CV and look for a new job, [and] found information out for us … which has given me the confidence to go back to my company … things were put in place at work, so that I could take my breaks when I needed them … It made a massive difference to me, personally … I was getting on board with [mental health condition], and now, I'm at work with renewed vigour.* (P9, Male, aged 59)

Long-term condition management

Weight reduction and increased fitness helped participants manage the pain and tiredness experienced as a result of their long-term health conditions. Some participants with diabetes noted being in better control of their condition due to reduced cholesterol and sugar levels. Accounts of positive improvements often followed years of worsening health and poor long-term condition management:

> *The instructor here has given me a programme that I'm working on to help build up the muscles for the legs and for my back … I'm managing [my arthritis] with the help of the gym … [also] my sugar levels … [have] come down to 4.9 … [and] my cholesterol was 3.1, which is good … all that had to do with Ways to Wellness and the exercise I've been doing.* (P6, Male, aged 67)

Long-term condition management services that service users were directed to by Link Workers were highlighted as extremely helpful, particularly the combination of expert and peer-led advice on coping and symptom management strategies:

> *The [name] group was wonderful because there were people there who had similar conditions … and they have medical professionals come in to talk about things that are specific to people with chest complaints … coping mechanisms … gentle exercises and things to help. It improves your stamina, your breathing abilities and everything. So yes, that has been absolutely wonderful … honestly it was the turning point in my life. Because I had reached rock bottom and I just needed practical help to try and get back up … you have to manage your expectations as well as your illness and it has helped considerably.* (P8, Female, aged 59)

For peer review only - http://bmjopen.bmj.com/site/about/guidelines.xhtml

For peer review only

Regularly engaging with services was challenging, particularly for people whose condition fluctuated and those suffering from more than one health problem. Service users worried about not always being able to attend which was often a reason for not sustaining engagement with services in the past:

> *I was really down, so I couldn't go on Friday. It's been like that since I first joined. I've been missing [sessions] because of my COPD … bad turns … chest infections … [but] they [Ways to Wellness] restarted me again, so they've looked after me … they've been really, really good … they've stuck with me.* (P1, Male, aged 70)

Participants experienced Ways to Wellness as an approach which recognised the long-term and complex nature of their health problems. It supported realistic, progressive and personalised goal-setting. Participants' expectations of progress were therefore achievable and reflected that a long-term approach was necessary to make improvements, helping people to live with, rather than cure, their conditions:

> *I am on the road but it is slow … work in progress … if it was easy I would have done it years ago … I have been well impressed [with Ways to Wellness]… because they have a very practical approach and know that it has got to be incremental … you can't do everything at once so you have got to start small and build up … they have got the big picture in mind but lots of little steps in between to get there … I would like to get back to being wonderful in the next five minutes it is not going to happen. So I know that I have got to take it bit by bit so it sticks.* (P8, Female, aged 59*)*

> *[Link Worker] epitomises the word, wellbeing, because that's what she's got in mind for you, your wellbeing. Nothing else, she gives the impression that you're the only one that she's looking after, at that time, in that moment in time, you're the only one that matters to her.* (P9, Male, aged 59)

DISCUSSION

This study provides evidence about the impact of social prescribing on health and wellbeing via improvements in health-related behaviours and long-term condition management. The specific model of social prescribing examined here comprised personalised support together with linkage into appropriate services facilitated by a Link Worker. The data reveal that key

For peer review only - http://bmjopen.bmj.com/site/about/guidelines.xhtml

elements of the model are that it is: long-term in nature; addresses the co-existence of multi-morbidity, mental health problems and social isolation; and, where applicable, tackles related socio-economic issues. This study demonstrates that the rapport and quality of the relationship between the Link Worker and service user was central to achieving wellbeing. Change in health-related behaviour and long-term condition management was facilitated through the use of setting realistic, progressive and personalised goals, problem solving, receiving regular feedback and social support. These behaviour change techniques have been shown to be effective in other lifestyle interventions. [23-25] Crucially these techniques were adopted whilst also supporting individuals to address social and economic problems. The intervention engendered feelings of control and self-confidence, reduced social isolation and led to positive physical changes such as weight loss, increased physical activity, improved long-term condition management and mental health, greater resilience and effective coping strategies to manage relapses.

Psychosocial problems, particularly mental health conditions and social isolation, are the most common reasons for referral into social prescribing interventions. [4 6] A review exploring the social prescribing evidence base identified 24 studies diverse in methodology and the service being evaluated. [6] The quantitative evidence in the review was limited with the exception of one randomised controlled trial demonstrating clinically important benefits in managing psychosocial needs at four months. [26] The quality of eight studies using quantitative methods was poor, with small samples and high drop-out rates.[6] Qualitative evidence, from this review and more broadly, also varies in quality, but generally indicates that those referred into social prescribing schemes report improved mental wellbeing and reduced social isolation. [6 9 27] A systematic review and meta-analysis of exercise referral schemes, a type of social prescribing intervention, targeted at sedentary individuals with or without a medical diagnosis was inconclusive with regards to increasing physical activity,

For peer review only - http://bmjopen.bmj.com/site/about/guidelines.xhtml

although the heterogeneity of the quality and the nature of the schemes is likely to have contributed to this conclusion.[28] The data presented in our study provide a much more in-depth account of *how* social prescribing positively influences long-term condition management and how this intervention can lead to improved physical and mental health outcomes.

Strengths, limitations and implications

The strength of this work lies in the depth and consistency of the service user accounts regarding the impact of intervention. The sample was relatively small, but data saturation was reached [16] and we interviewed people with a wide range of long-term conditions, almost all with multi-morbidity, and many experiencing social isolation and mental health problems. Methodologically, we followed accepted practice in fieldwork, analysis and interpretation. [29] Participants were those who agreed to a referral, then enrolled into and remained with Ways to Wellness. Although we know that some study participants were initially reluctant to engage with Ways to Wellness, we cannot make claims about patients with long-term conditions who did not engage in the first place or who dropped out of the programme early. This is undoubtedly an important group for further research. Our findings may be transferable [30] to other similar populations in socially disadvantaged areas, if and where a similar intervention is available.

Long-term health conditions are arguably the greatest challenge facing the NHS; they have an adverse effect on quality of life and reduce life expectancy. Multi-morbidity accounts for over 70 percent of total healthcare spend. [31] Non-medical interventions are proposed as a cost-effective alternative to foster self-management in people with long-term conditions. [32 33] People from lower socio-economic groups experience higher levels of chronic disease, and also have poorer condition management, worse health outcomes, and higher mortality. [31]

For peer review only

Behavioural risk factors for long-term conditions include poor diet, smoking and physical inactivity, all of which are socioeconomically patterned. [34] However, lifestyle behaviours are also affected by wider health determinants and are not simply choices that individuals make. [35 36] Supporting people to make meaningful and sustainable lifestyle changes is complex, time consuming, and a challenge for time-limited GPs. [11] Given the complex and long-term nature of the problems faced by service users, it is unsurprising that a holistic and relatively intensive approach, as offered by Ways to Wellness, is required in order to facilitate and maintain behaviour change, and it is clearly not possible, nor appropriate to offer this level of support routinely in primary care. Referral into social prescribing programmes, is likely to continue due to the need to tackle long-term health conditions, multi-morbidity and an ageing population in the face of stretched NHS resources. [33] [37] The range of approaches to, and models of, social prescribing [38] which makes it challenging to amass good quality evidence to inform the commissioning of social prescribing. [37 38] In order to inform practitioners and commissioners, future research should be robust and comparative, addressing when, for whom and how well the programme works, include validated measures of intervention impact and accurate costs. [37] To this we would add further qualitative, particularly ethnographic, research to examine potential wider impacts on families and communities and how community-based, non-medical and personalised interventions supplement health care.

For peer review only - http://bmjopen.bmj.com/site/about/guidelines.xhtml

Acknowledgements

We thank the participants for sharing their experiences, Link Workers and Link Worker Managers at Mental Health Concern, First Contact Clinical, Healthworks and Changing Lives for their assistance with recruitment, and Chris Drinkwater, Tara Case and Amie Callan from Ways to Wellness for their support.

Contributors

SM, LP and NO'B conceived and designed the research and obtained funding. MS and SL undertook interviews. SM, MS, LP and SL and contributed to the data analysis. SM, MS, SL, LP and NO'B wrote the paper. All authors read and approved the final version.

Funding

This work was funded by the Cabinet Office of the UK Government (grant number A1543). The researchers are independent of the funders and the views and opinions expressed in this paper are those of the authors and not necessarily those of the Cabinet Office. The funders and sponsors (Newcastle University) had no role in the design, collection, analysis and interpretation of the data, or the writing of this paper or the decision to submit this article for publications. All authors had full access to the data in the study and can take full responsibility for the integrity of the data and the accuracy of the analysis.

Competing interests

None

For peer review only - http://bmjopen.bmj.com/site/about/guidelines.xhtml

Ethics approval

This study was approved by Newcastle University Faculty of Medical Sciences Research Ethics Committee (00868/2014)

Data sharing statement

No additional data are available

For peer review only

For peer review only - http://bmjopen.bmj.com/site/about/guidelines.xhtml

References

1. Local Government Association. Just what the doctor ordered. Social Prescribing - a guide for local authorities. http://wwwlocalgovuk/documents/10180/7632544/L16-108+Just+what+the+doctor+ordered+-+social+prescribing+-+a+guide+to+local+authorities/f68612fc-0f86-4d25-aa23-56f4af33671d, 2016.
2. Social Prescribing Network. Report of the Annual Social Prescribing Network Conference. London: University of Westminster, 2016.
3. South J, Higgins TJ. Can social prescribing provide the missing link? *Primary Health Care Research and Development* 2008;9:310 - 18.
4. Mossabir R, Morris R, Kennedy A, et al. A scoping review to understand the effectiveness of linking schemes from healthcare providers to community resources to improve the health and well-being of people with long-term conditions. *Health & Social Care in the Community* 2015;23(5):467-84. doi: 10.1111/hsc.12176
5. Popay J, Kowarzik U, Mallinson S, et al. Social problems, primary care and pathways to help and support: addressing health inequalities at the individual level. part I: the GP perspective. *Journal of Epidemiology and Community Health* 2007;61(11):966-71. doi: 10.1136/jech.2007.061937
6. Kilgarriff-Foster A, O'Cathain A. Exploring the components and impact of social prescribing. *Journal of Public Mental Health* 2015;14(3):127-34.
7. Kimberlee R. What is the value of social prescribing? *Advances in Social Sciences Research Journal* 2016;3(3):29-35.
8. Dayson C, Bashir N. The social and economic impact of the Rotherham Social Prescribing Pilot. In: Centre for Regional Economic and Social Research SHU, ed. https://www4.shu.ac.uk/research/cresr/sites/shu.ac.uk/files/social-economic-impact-rotherham.pdf, 2014.
9. Kimberlee R, Ward R, Jones M, et al. Proving our values: Measuring the economic impact of Well Spring Healthy LIving Centre's Social Prescribing for low level mental health isues encountered by GP services. Bristol: University of the West of England, 2014.
10. Husk K, Blockley K, Lovell R, et al. What approaches to social prescribing work, for whom, and in what circumstances? A protocol for a realist review. *Systematic reviews* 2016;5(1):93-93. doi: 10.1186/s13643-016-0269-6
11. Brandling J, House W. Social prescribing in general practice: adding meaning to medicine. *British Journal of General Practice* 2009;59(563):454-56. doi: 10.3399/bjgp09X421085
12. Ways to Wellness [Available from: http://waystowellness.org.uk/ accessed 14/11/16.
13. NHS North East Innovation Fund. Year of Care, Thanks for the Petunias- a guide to developing and commissioning non-traditional providers to support the self management of people with long term conditions, 2011.
14. Department for Communities and Local Government. The English Indices of Deprivation 2010, 2013.
15. Newcastle West Clinical Commissioning Group. Annual Report and Accounts 2013-14,. Newcastle, 2014.
16. Denzin NK, Lincoln YS. Handbook of qualitative research. London: Sage 2000.
17. Green J, Thorogood N. Qualitative Methods for Health Research. London: Sage 2014.
18. NVivo 10. Computer Assisted Software for Qualitative Analysis 2010 [accessed 20.3.07 2007.
19. Glaser B, Strauss A. The Discovery of Grounded Theory. Chicago: Aldine 1967.
20. Silverman D. Doing qualitative research. London: Sage 2000.
21. Barbour RS. Checklists for improving rigour in qualitative research: a case of the tail wagging the dog? *BMJ : British Medical Journal* 2001;322(7294):1115-17.
22. ONS. SOC2010 volume 3: the National Statistics Socio-economic classification (NS-SEC rebased on SOC2010) 2010 [Available from:

For peer review only - http://bmjopen.bmj.com/site/about/guidelines.xhtml

http://webarchive.nationalarchives.gov.uk/20160105160709/http://www.ons.gov.uk/ons/guide-method/classifications/current-standard-classifications/soc2010/soc2010-volume-3-ns-sec--rebased-on-soc2010--user-manual/index.html accessed 14/11/16.
23. Greaves C, Sheppard K, Abraham C, et al. Systematic review of reviews of intervention components associated with increased effectiveness in dietary and physical activity interventions. *BMC Public Health* 2011;11(1):119.
24. Lara J, Evans EH, O'Brien N, et al. Association of behaviour change techniques with effectiveness of dietary interventions among adults of retirement age: a systematic review and meta-analysis of randomised controlled trials. *BMC Medicine* 2014;12 doi: 10.1186/s12916-014-0177-3
25. O'Brien N, McDonald S, Araujo-Soares V, et al. The features of interventions associated with long-term effectiveness of physical activity interventions in adults aged 55-70 years: a systematic review and meta-analysis. *Health Psychology Review* 2015;9(4):417-33. doi: 10.1080/17437199.2015.1012177
26. Grant C, Goodenough T, Harvey I, et al. A randomised controlled trial and economic evaluation of a referrals facilitator between primary care and the voluntary sector. *BMJ* 2000;320:419-23.
27. City and Hackney Clinical Commissioning Group and University of East London. Shine 2014 final report: Social prescribing: integrating GP and community assets for health, 2015.
28. Pavay TG, Taylor AH, Fox KR, et al. Effect of exercise referral schemes in primary care on physical activity and improving health outcomes: systematic review and meta-analysis. *BMJ* 2011;343:d6462.
29. Barbour R, S. The Newfound Credibility of Qualitative Research? Tales of Technical Essentialism and Co-Option. *Qualitative Health Research* 2003;13(7):1019-27.
30. Ritchie J, Lewis J. Qualitative Research Practice. A Guide for Social Scientists. London: Sage 2003.
31. Barnett K, Mercer SW, Norbury M, et al. Epidemiology of multimorbidity and implications for health care, research, and medical education: a cross-sectional study. *The Lancet* 2012;380(9836):37-43.
32. Department of Health. Our health, Our Care, Our Say: A New Direction for Community Services. In: Health Do, ed.: TSO, 2006:1-236.
33. Coulter A, Roberts S, A. D. Deliversing better servgices for people with long-term contitions: building the house of care. London: The King's Fund, 2013.
34. Wardle J, Steptoe A. Socioeconomic differences in attitudes and beliefs about healthy lifestyles. *Journal of Epidemiology and Community Health* 2003;57(6):440-43. doi: 10.1136/jech.57.6.440
35. UCL Institute of Health Equity. Tackling Health Inequalities Through Action on the Social Determinants of Health http://www.instituteofhealthequity.org/projects/tackling-health-inequalities-through-action-on-the-social-determinants-of-health-lessons-from-experience 2014 [
36. Salmon P, Hall GM. Patient empowerment and control: a psychological discourse in the service of medicine. *Social Science & Medicine,* 2003;57:1969-80.
37. Centre for Reviews and Dissemination. Evidence to inform the commissioning of social prescribing. York: The University of York, https://www.york.ac.uk/media/crd/Ev%20briefing_social_prescribing.pdf, 2015.
38. Kimberlee R. What is social prescribing. *Advances in Social Sciences Research Journal* 2015;2(102-110)

For peer review only - http://bmjopen.bmj.com/site/about/guidelines.xhtml

Coreq Checklist for Manuscript ID bmjopen-2016-015203 entitled "Social prescribing to improve health and wellbeing for people with long-term conditions: qualitative study of service user perceptions"

**Domain 1: Research team and reflexivity**			**Authors' response**
1.	Interviewer/facilitator	Which author/s conducted the interview or focus group?	Page 9, MS and SL conducted interviews
2.	Credentials	What were the researcher's credentials? *E.g. PhD, MD*	Page 1<br>SM PhD<br>MS PhD<br>SL BA (Hons)<br>LP PhD<br>NOB PhD
3.	Occupation	What was their occupation at the time of the study?	SM Senior Lecturer<br>MS Research Associate<br>SL Research Associate<br>LP Research Associate<br>NOB Research Methodologist
4.	Gender	Was the researcher male or female?	Page 1 Female
5.	Experience and training	What experience or training did the researcher have?	MS PhD training<br>SL extensive undergrad and post grad experience

For peer review only

Relationship with participants			
6.	Relationship established	Was a relationship established prior to study commencement?	Pages 8-9<br><br>No
7.	Participant knowledge of the interviewer	What did the participants know about the researcher? e.g. *personal goals, reasons for doing the research*	Pages 8-9<br><br>Reasons for doing the research
8.	Interviewer characteristics	What characteristics were reported about the interviewer/facilitator? e.g. *Bias, assumptions, reasons and interests in the research topic*	Pages 8-9<br><br>Interest in the impact of the intervention
**Domain 2: study design**			
Theoretical framework			
9.	Methodological orientation and Theory	What methodological orientation was stated to underpin the study? *e.g. grounded theory, discourse analysis, ethnography, phenomenology, content analysis*	Page 9<br><br>Thematic analysis
Participant selection			

For peer review only

10.	Sampling	How were participants selected? e.g. *purposive, convenience, consecutive, snowball*	Page 8<br><br>Purposive
11.	Method of approach	How were participants approached? e.g. *face-to-face, telephone, mail, email*	Page 8<br><br>Participants were approached initially by gatekeepers (Link Workers). Once willing to participate, researchers contacted participants by telephone
12.	Sample size	How many participants were in the study?	Page 10<br><br>N=30
13.	Non-participation	How many people refused to participate or dropped out? Reasons?	Page 8<br><br>As we used gatekeepers to contact participants, we do not have accurate numbers of how many people refused to participate, but anecdotally, most who were approached were willing to take part.<br><br>Following agreement to take part in the study, no participants dropped out.
Setting			
14.	Setting of data collection	Where was the data collected? e.g. *home, clinic, workplace*	Page 8-9<br><br>Most interviews took place in participants' homes, but some took place at a community venue.

For peer review only

15.	Presence of non-participants	Was anyone else present besides the participants and researchers?	No
16.	Description of sample	What are the important characteristics of the sample? *e.g. demographic data, date*	Page 11, Table 1
Data collection			
17.	Interview guide	Were questions, prompts, guides provided by the authors? Was it pilot tested?	Page 8.<br><br>Topic guide developed with prompts on: referral procedures; level and type of engagement with Ways to Wellness; goal-setting; linkage to other services; long-term condition management; changes resulting from involvement with Ways to Wellness; and views of the service.<br><br>Not pilot tested with participants.
18.	Repeat interviews	Were repeat interviews carried out? If yes, how many?	No
19.	Audio/visual recording	Did the research use audio or visual recording to collect the data?	Page 9<br><br>Data were digitally recorded
20.	Field notes	Were field notes made during and/or after the	Page 9

For peer review only

		interview or focus group?	Fieldnotes were made immediately after each interview and shared amongst the team.
21.	Duration	What was the duration of the interviews or focus group?	Page 9<br><br>Duration ranged between eight minutes and one hour 27 minutes (average 41 minutes)
22.	Data saturation	Was data saturation discussed?	Page 9<br><br>Interviews continued until data saturation was reached.
23.	Transcripts returned	Were transcripts returned to participants for comment and/or correction?	No
**Domain 3: analysis and findings**z			
Data analysis			
24.	Number of data coders	How many data coders coded the data?	Page 9<br><br>Two coders (MS, SL)
25.	Description of the coding tree	Did authors provide a description of the coding tree?	No
26.	Derivation of themes	Were themes identified in advance or derived from the	Page 9<br><br>Some a priori themes based on topic guide

For peer review only

		data?	identified in advance, but further themes emerged from the data
27.	Software	What software, if applicable, was used to manage the data?	Page 9<br><br>NVIVO
28.	Participant checking	Did participants provide feedback on the findings?	No
Reporting			
29.	Quotations presented	Were participant quotations presented to illustrate the themes / findings? Was each quotation identified? e.g. *participant number*	Pages 14-20<br><br>Participant quotes presented to illustrate themes, all participants identified by number, gender and age.
30.	Data and findings consistent	Was there consistency between the data presented and the findings?	Pages 14-20 (results) and Page 20-21 discussion<br><br>Yes
31.	Clarity of major themes	Were major themes clearly presented in the findings?	Pages 14-20<br><br>Major themes presented as:<br><br>Impact of long-term conditions and multi-morbidity<br><br>Link Worker Roles<br><br>Positive Impact of the Intervention
32.	Clarity of minor	Is there a description of diverse cases or	Page 22

For peer review only

	themes	discussion of minor themes?	Participants accounts were highly consistent, although data interrogated for 'diverse/deviant' cases.

For peer review only

BMJ Open

Link worker social prescribing to improve health and wellbeing for people with long-term conditions: qualitative study of service user perceptions

Journal:	*BMJ Open*
Manuscript ID	bmjopen-2016-015203.R2
Article Type:	Research
Date Submitted by the Author:	07-Apr-2017
Complete List of Authors:	Moffatt, Suzanne; Newcastle University, Institute of Health and Society<br>Steer, Mel; Newcastle University, Institute of Health and Society<br>Lawson, Sarah; Newcastle University, Institute of Health and Society<br>Penn, Linda; Newcastle University, Institute of Health and Society<br>O'Brien, Nicola; Newcastle University, Institute of Health and Society
**Primary Subject Heading**:	Public health
Secondary Subject Heading:	Qualitative research
Keywords:	Social prescribing, Long-term condition management, Health inequalities, Multi-morbidity

SCHOLARONE™ Manuscripts

For peer review only

Link Worker social prescribing to improve health and wellbeing for people with long-term conditions: qualitative study of service user perceptions.

Suzanne Moffatt, PhD[1]

Mel Steer, PhD

Sarah Lawson, BA (Hons)

Linda Penn, PhD

Nicola O'Brien, PhD

Word count: 3799 (excluding table, quotes and references)

Word count: 6496 (including tables and quotes, excluding references)

1.Corresponding author

Suzanne Moffatt

Senior Lecturer in Social Gerontology

Institute of Health and Society

Newcastle University

Campus for Ageing and Vitality

Biomedical Research Building

Newcastle upon Tyne, NE4 5PL

Email: suzanne.moffatt@ncl.ac.uk

Tel +44 (0) 191 208 5005

For peer review only - http://bmjopen.bmj.com/site/about/guidelines.xhtml

For peer review only

ABSTRACT

Objectives: To describe the experiences of patients with long-term conditions who are referred to and engage with a Link Worker social prescribing programme and identify the impact of the Link Worker programme on health and wellbeing.

Design: Qualitative study using semi-structured interviews with thematic analysis of the data.

Intervention: Link Worker social prescribing programme comprising personalised support to identify meaningful health and wellness goals, ongoing support to achieve agreed objectives and linkage into appropriate community services.

Setting: Inner-city area in West Newcastle upon Tyne, UK (population N=132,000) ranked 40th most socio-economically deprived in England, served by 17 general practices.

Participants: Thirty adults with long-term conditions, 14 female, 16 male aged 40-74, mean age 62 years, 24 White British, 1 White Irish, five from Black and Minority Ethnic communities.

Results: Most participants experienced multi-morbidity combined with mental health problems, low self-confidence and social isolation. All were adversely affected physically, emotionally and socially by their health problems. The intervention engendered feelings of control and self-confidence, reduced social isolation and had a positive impact on health-related behaviours including weight loss, healthier eating and increased physical activity. Management of long-term conditions and mental health in the face of multi-morbidity improved and participants reported greater resilience and more effective problem solving strategies.

Conclusions: Findings suggest that tackling complex and long-term health problems requires an extensive holistic approach not possible in routine primary care. This model of social

For peer review only - http://bmjopen.bmj.com/site/about/guidelines.xhtml

prescribing, which takes into account physical, mental health, social and economic issues, was successful for patients who engaged with the service. Future research on a larger scale is required to assess when and for whom social prescribing is clinically and cost effective.

For peer review only

For peer review only - http://bmjopen.bmj.com/site/about/guidelines.xhtml

Strengths and limitations of this study

- This is the first UK study to provide detailed insight into the impact of a Link Worker social prescribing programme on health and wellbeing among adults with long-term conditions living in an area of high socio-economic deprivation.
- Our interview data showed that the Link Worker social prescribing programme engendered feelings of control and self-confidence, reduced social isolation and led to positive physical and behavioural changes such as weight loss, increased physical activity, improved mental health and long-term condition management as well as greater resilience and effective coping strategies to manage relapses.
- Key elements of the Link Worker social prescribing model are that it: addresses the co-existence of multi-morbidity, mental health problems and social isolation; is long-term in nature; and, where applicable, tackles related socio-economic issues.
- A strength of this work was the depth and consistency of participant accounts regarding the impact of the programme. Limitations were that we did not capture the experiences of patients with long-term conditions who refuse a referral or who drop out of the programme early, nor were we able to collect information on the frequency with which participants engaged in activities they were referred to.
- Our study adds to the emerging evidence base on Link Worker social prescribing by demonstrating improvements in health-related behaviours and long-term condition management.

For peer review only - http://bmjopen.bmj.com/site/about/guidelines.xhtml

BACKGROUND

Social prescribing enables health care practitioners to refer patients to a range of non-clinical services. [1] Primarily, but not solely, directed at people with long-term conditions, social prescribing harnesses assets within the voluntary and community sector to improve and encourage self-care and facilitate health-creating communities. [2 3 4] There is increasing interest in social prescribing as a means of addressing complex health, psychological and social issues presented in primary care, as well as its potential to reduce health inequalities.[5] A recent review of social prescribing indicates that, despite a small and largely inconclusive evidence base, there is the potential for credible psychosocial benefits to be incurred by patients with mental health problems, and for health and wellbeing improvements to be seen in people with long-term conditions.[6] Whilst less attention has been paid to the impact of social prescribing on physical health and resource use, [4] improvements in physical activity, [7] reductions in hospital resource use, [8] and GP attendance [9] have been attributed to social prescribing, although longer-term studies with larger sample sizes are required for more definitive evidence. [4]

As yet there is no agreed definition of social prescribing, [2] although there is broad consensus that it helps patients to access non-clinical sources of support, predominantly in the community sector,[3] and is a means to address the well-documented social and economic factors that accompany long-term illness beyond the healthcare setting.[4] In the UK, social prescribing has been taking place on a small scale for a number of years and there are several operating models.[10] These models vary in two ways; the actual activities or services offered and the level of support given to patients following referral. Recognising that patients who are simply given information about a service will not necessarily take it up, most schemes involve a 'facilitator' coupled with personal support,[11] although the level of ongoing support offered varies considerably. Services into which patients are referred vary, and can include

For peer review only - http://bmjopen.bmj.com/site/about/guidelines.xhtml

activities that involve physical activity such as gyms, walking groups, gardening or dance clubs; weight management and healthy eating activities, such as cooking clubs. Addressing wider economic and social issues can involve referral into services which address welfare, debt, housing and employment issues. Groups, such as those targeted at people with specific long-term conditions, for example diabetes, chronic obstructive pulmonary disease, may also be accessed via social prescription. Our definition concurs with that of the Social Prescribing Network of Ireland and Great Britain, "enabling healthcare professionals to refer patients to a link worker, to co-design a non-clinical social prescription to improve their health and wellbeing" and use services provided by the voluntary and community sector. (p19) [2]

Ways to Wellness [12] is one of the first UK organisations to deliver social prescribing on a large and prolonged scale; funded for seven years through a social impact bond model, with an overall target of 11,000 users over this period. Based in West Newcastle upon Tyne, an area with some of the most socio-economically deprived wards in England, Ways to Wellness covers 17 general practices. Referral criteria are men and women aged 40-74 with one or more of the following long-term conditions: diabetes (type 1&2), chronic obstructive pulmonary disease, asthma, coronary heart disease, heart failure, epilepsy, osteoporosis, with or without anxiety or depression. Ways to Wellness is delivered by four voluntary sector organisations.

Following extensive consultation with patients and health care professionals over an eight year period,[13] Ways to Wellness provides a 'hub' model of social prescribing in which a Link Worker trained in behaviour change methods offers a holistic and personalised service. Following referral from a primary care practitioner (GP, practice nurse, health care assistant), meaningful health and wellness goals are jointly identified and service users are connected,

For peer review only - http://bmjopen.bmj.com/site/about/guidelines.xhtml

when desired, to community and voluntary groups and resources. The service comprises: (a) individual assessment, motivational interviewing and action planning; (b) completion of an initial 'Well-being Star' assessment and subsequent Well-being Star assessments every six months thereafter for the duration of the patient's involvement; (c) help to access community services (e.g. welfare rights advice, walking groups, physical activity classes, arts groups, continuing education); (d) promotion of volunteering opportunities, and; (e) promotion of improved self-care and sustained behaviour change related to healthier lifestyle choices. Thus, the programme is highly individualised with patient engagement varying in terms of intensity, duration, personalised goal-setting and onward referral. Patients can remain with the programme for up to two years, but with Link Worker discretion beyond two years if required; frequency of contact with the Link Worker is mutually agreed, varies between and within patients depending on current need and circumstances, and can be face to face, via telephone, email and/or text message. Data for this study were collected in the first 14 months of the Link Worker social prescribing programme implementation.

This qualitative study aimed to capture the experiences of patients engaged with Ways to Wellness in its first 14 months of operation and to identify the impact of the Link Worker social prescribing programme on health and wellbeing.

For peer review only

For peer review only - http://bmjopen.bmj.com/site/about/guidelines.xhtml

METHODS

Setting

This study was set in an inner-city area of high socio-economic deprivation in the west of Newcastle upon Tyne (population n=132,000), ranked among the 40 most deprived in England according to the Index of Multiple Deprivation.[14] Eighteen per cent of residents have long-term conditions and receive sickness and disability-related benefits, which is higher than the national average.[15]

Recruitment and sampling

The four Ways to Wellness provider organisations acted as gatekeepers and approached service users on our behalf, explaining the study and issuing a participant information sheet. Those who agreed for their details to be passed on to the researcher were contacted by telephone to ascertain willingness to participate, and, if willing, an interview was arranged. We set out to obtain a maximum variation sample across the four provider organisations based on the following criteria: age, gender, long-term condition, marital status, employment status, socio-economic status, and level of engagement with Ways to Wellness (intensive to non-intensive). For the purposes of sampling, long-term condition was ascertained by the Link Worker via the referral form, and had therefore been diagnosed by a GP or other health care professional. The recruitment period was January to June 2016. Ways to Wellness was operational from April 2015, and in the 14 months since it started, 864 women and 739 men, average age 59, were referred from primary care and attended at least one Link Worker session.

Data collection

We undertook one semi-structured interview with each participant between January and June 2016 whilst they were engaged with Ways to Wellness (length of engagement ranged from 4-

For peer review only - http://bmjopen.bmj.com/site/about/guidelines.xhtml

14 months). A topic guide was developed covering: referral procedures; level and type of engagement with Ways to Wellness; goal-setting; linkage to other services; long-term condition management; changes resulting from involvement with Ways to Wellness; and views of the service. Interviews took place in participants' homes or an alternative venue of their choosing (often where they attended their Ways to Wellness appointment). Interviews were carried out by two researchers (MS and SL). Following consent procedures, demographic details were collected. Interviews continued until consistencies were identified across participants and data.[16]

Transcription, data management and analysis

Interviews lasted between eight minutes and one hour 27 minutes (average 41 minutes), were digitally recorded and transcribed verbatim. Fieldnotes were made immediately after each interview and shared amongst the team. Transcripts were anonymised and checked against recordings for accuracy. Thematic analysis was used [17] with data management supported by NVivo10 software. [18] Following close reading of the transcripts, a coding scheme was developed which contained a-priori themes based on the topic guide as well as further themes which emerged from the data. The scheme captured data relating to: referral, multi-morbidity, experiences of Ways to Wellness service delivery and onward referral, relationship with the Link Worker, impact of Ways to Wellness and barriers to service engagement. The coding framework was applied to an initial randomly selected five interviews, which were double-coded by MS and SL. Following this, the coding frame was reviewed by all team members, modifications agreed and made before being applied to all interviews. Line-by-line coding and constant comparison were used to code the entire dataset;[19 20] deviant case analysis, where we sought out opinions which modified or contradicted the analysis, was used to enhance validity.[21]

For peer review only - http://bmjopen.bmj.com/site/about/guidelines.xhtml

RESULTS

The impact of Ways to Wellness is described by our detailed analysis of the following three themes: negative impact of long-term conditions and multi-morbidity; Link Worker roles; and positive impact of the programme.

Participant characteristics

As shown in Table 1, 30 adults, 14 women and 16 men, aged 40-74 years (mean age 62 years) took part. Thirteen participants were over state pension age; four of the 17 participants of working age were in employment. Occupational social class [22] based on current or previous main employment indicates that the sample included individuals from across the social class spectrum, excepting social class 1; social classes 2-4 accounted for two thirds of the sample and the remaining third were from social classes 5-8. Five participants were from Black and Minority Ethnic Communities, one identified as White Irish and the remaining 24 were White British. Multi-morbidity was a prominent feature of the sample. Based on self-reported health conditions, only one participant (15) had a single long-term condition. Most participants had more than one 'referral' long-term condition, had other health problems and associated mental health issues, low confidence and social isolation. With the exception of the diagnosed long-term conditions that triggered a referral to Ways to Wellness (i.e. diabetes (type 1&2), chronic obstructive pulmonary disease, asthma, coronary heart disease, heart failure, epilepsy, osteoporosis) other physical and mental health problems were self-reported at interview. No further assessment of physical or mental health was made during the study.

At the time of interview, participants had been receiving the social prescribing programme for between four and fourteen months. Table 1 shows that the number of services that each participant reported being linked into ranged from zero to five; the average number was 1.7. However, seven participants (11, 12, 13, 16, 24, 25, 30) obtained welfare benefits advice

For peer review only - http://bmjopen.bmj.com/site/about/guidelines.xhtml

from their Link Workers, rather than being linked into welfare rights services, demonstrating that some Link Workers drew on their specialist knowledge to assist their client rather than make a referral. Table 2 shows that 54 referrals to community, voluntary and NHS services were made for the 30 participants. These are categorised as follows: long-term condition management; mental health; physical activity; weight management and healthy eating; NHS services (e.g. physiotherapy), welfare rights advice (e.g. benefits advice, aids and adaptations), learning/employment assistance (e.g. CV writing), voluntary work, arts based activities (e.g. choir, art therapy), community based activities (e.g. gardening, fishing, crafts). Services promoting physical activity were the most common linkage.

For peer review only

For peer review only - http://bmjopen.bmj.com/site/about/guidelines.xhtml

Table 1: Demographic characteristics and long-term health conditions of study participants

ID	Sex	Employment status	Occupational social class [a,b,c,d]	No. of WtW conditions[e]	Total no. of non-WtW conditions[f]	Mental health / social isolation[g]	Age band	Months involved with WtW at interview	No. of services linked into
1	Male	Retired	7	3	3	Yes	70-74	4	2
2	Female	Retired	2	3	3	Yes	70-74	4	3
3	Female	Employed	7	1	1	Yes	45-49	5	1
4	Female	Unemployed	8	2	1	Yes	55-59	5	2
5	Female	Retired	2	2	4	Yes	65-69	6	1
6	Male	Retired	4	2	1	Yes	65-69	7	0
7	Male	Unemployed	7	2	1	No	55-59	7	1
8	Female	Unemployed	2	2	3	No	55-59	7	2
9	Male	Employed	4	3	3	Yes	55-59	7	5
10	Male	Unemployed	4	2	4	Yes	60-64	7	4
11	Male	Unemployed	2	2	4	Yes	45-49	7	5[i]
12	Male	Unemployed	4	1	3	Yes	55-59	7	1[i]
13	Male	Unemployed	7	1	2	Yes	60-64	7	0[i]
14	Male	Unemployed	2	2	1	No	60-64	7	5
15	Female	Retired	8	1	0	No	70-74	8	3
16	Female	Retired	2	2	0	No	65-69	8	0[i]
17	Female	Unemployed	3	2	0	Yes	50-54	9	1
18	Female	Retired	3	3	2	Yes	65-69	10	1
19	Male	Retired	6	2	4	Yes	65-69	10	2
20	Male	Retired	4	2	1	No	70-74	11	1
21	Female	Retired	5	3	1	No	70-74	12	3
22	Female	Unemployed	3	2	2	Yes	40-44	12	1
23	Male	Retired	6	2	4	No	70-74	12	2
24	Female	Retired	2	2	0	Yes	70-74	12	0[i]
25	Female	Unemployed	2	3	1	Yes	50-54	12	0[i]
26	Male	Retired	3	2	3	No	70-74	13	3
27	Male	Employed	4	1	1	Yes	40-44	14	0
28	Male	Retired	4	2	1	No	70-74	14	3
29	Male	Employed	6	1	3	Yes	60-64	NK[h]	1
30	Female	Unemployed	2	2	2	Yes	45-49	NK[h]	1[i]

For peer review only - http://bmjopen.bmj.com/site/about/guidelines.xhtml

a. SOC2010 volume 3: the National Statistics Socio-economic classification (NS-SEC rebased on SOC2010) http://webarchive.nationalarchives.gov.uk/20160105160709/http://www.ons.gov.uk/ons/guide-method/classifications/current-standclassifications/soc2010/soc2010-volume-3-ns-sec--rebased-on-soc2010--user-manual/index.html . Last accessed 30 August 2016.

b. Last occupation used except when had changed due to health issues and occupation before health forced change was used for classification.

c c. Occupations of NS-Sec analytical class coded using classification tool found at: https://www.ons.gov.uk/methodology/classificationsandstandards/standardoccupationalclassificationsoc/soc2010. Last accessed 21 September 2016.

d. 1 =2 = Lower managerial, administrative and professional occupation. 3 = Intermediate occupation. 4 = Small employers and own account workers. 5 = Lower supervisory and technical occupation. 6. Semi-routine occupation. 7 =Routine occupation. 8 = Never worked / long-term unemployed.

e. Conditions which triggered a referral to Ways to Wellness were: diabetes (type 1&2), chronic obstructive pulmonary disease, asthma, coronary heart disease, heart failure, epilepsy, osteoporosis) with or without anxiety or depression. This column indicates the number of 'Ways to Wellness' long-term conditions that participants had been diagnosed with by a medical practitioner.

f. Based on self-report at interview.

g. A broad category that includes low mood, anxiety, depression, loneliness and social isolation and is based on self-report at interview where participants described or reported these conditions or feelings.

h. Not known

i. Received welfare benefits advice from Link Worker and were not referred onto specialist welfare rights services.

For peer review only - http://bmjopen.bmj.com/site/about/guidelines.xhtml

Table 2 Number of onward referrals by service category amongst interview sample

Category	Number of referral to other services
Long term condition management (e.g. voluntary sector support groups)	7
Mental health (e.g. CBT)	1
Physical activity (e.g. gym, walking group, swimming)	21
Weight management/healthy eating	5
NHS services (e.g. physiotherapy)	7
Welfare rights advice (e.g. benefits advice, aids and adaptations)	3
Learning/Employment assistance (e.g. CV writing)	5
Voluntary work	1
Arts based activities (e.g. choir, art therapy)	2
Community based activities (e.g. gardening, fishing, crafts)	2

For peer review only

Impact of long-term conditions and multi-morbidity

All participants had been deeply affected, physically, emotionally and socially by their health problems. Physical effects included pain, sleep problems, side effects of medication, continence issues and significant functional limitations. With increasing age, existing conditions worsened and multi-morbidity increased:

> *I've got COPD, high blood pressure, diabetic … a prolapsed disc and … an operation on it that didn't work, so I'm in constant pain with my back. Just generally not fit, not well … stent put in because I had kidney stones. They were supposed to take it out … but because I had a temperature they didn't take it out. They left it in, but I'm having nothing but trouble with it, water infections, feeling as if I want to go to the toilet all the time and when I do go I don't pass anything. It's like dying for a wee all the time … It's got me really down.* (P1, Male, 70-74)

Some of those experiencing depression reported that this had been with them for many years, but most accounts of depression and/or anxiety were attributed to their long-term condition(s) and the difficulties of carrying out a 'normal' life. Wider participation within family and community networks was affected, as well as basic activities of daily living such as shopping or housework. Social isolation was common amongst many of the participants, but for those of working age, unemployment, lowered incomes and reliance on sickness welfare benefits were commonplace. The overall picture was of lives seriously blighted by long-term health problems:

> *I want to get back to work. I was used to doing things and it is really hard not being able to do all the things I used to do, yes, and I was depressed at the time as well … it looks like a long road to get back to being healthy again; and I needed all the help I could get … I have got asthma and I have got diabetes and high blood pressure. And since I have had the treatment for cancer… I was fine after the operation but it was the radiotherapy that really knocked me out … I mean they do warn you that it affects your energy – but my energy levels just plummeted and I wasn't able to do hardly anything.* (P8, Female, 55-59)

For peer review only - http://bmjopen.bmj.com/site/about/guidelines.xhtml

Link Worker roles

Connecting with service users

Link Workers operated an accessible, flexible service between 9am and 5pm, Monday-Friday. Following primary care referral, Link Workers contacted service users by telephone to arrange an initial appointment, which could be held at GP practices, community centres, cafés, or infrequently, participants' homes. In general, contact with Link Workers was more frequent during the initial stages, reducing as the relationship developed and the service user became more independent; however, contact also decreased or increased with current need. Face-to-face contact was also supplemented by telephone, email or text:

> *[Link Worker] says, "I'm there, basically, any time," obviously within working hours, but she says, "Just phone me up if you need me, and any questions or anything." So basically I can see her as often as I want or as little as I want, but she likes me to keep her informed of anything happening, so she knows.* (P11, Male, 45-49)

Participants appreciated the flexibility and 'open door' nature of Ways to Wellness, although this could be limited for those who were working. Of particular value was the potential to be engaged with the service for up to two years. Due to the long-term and complex nature of conditions which often fluctuated, participants recognised that a shorter term approach would be inadequate, particularly when accounting for wider life events such as job loss or bereavement:

> *Well, I think you can dip in and dip out. It's the kind of thing if you need them, you phone them and they'll get straight back to you. They're there, I know they're there … if something happens to me now.* (P13, Male, 60-64)

Link Worker approach

Participants consistently reported feeling at ease and relaxed with their Link Worker, which enabled them to develop an open and trusting relationship. The Link Workers' person-

For peer review only - http://bmjopen.bmj.com/site/about/guidelines.xhtml

centred and non-judgemental approach facilitated trust, feelings of control and a readiness to reflect on current circumstances and implement positive changes. Service users felt listened to and valued:

> *… [Link Worker] really helped me along … they're not judgemental … and they were so easy going and they were just lovely.* (P17, Female, 50-54)

> *She [Link Worker] just brought things up … we'd have a discussion … we just started talking and that's when I just came out with different things … and she gave the feeling that you could open up to her … where some people, you are guarded because you don't want them to know too much of your private life.* (P9, Male, 55-59)

A central part of the Link Worker role was to facilitate engagement with other services, as shown in Table 2. The level and type of support offered to facilitate engagement varied, and was balanced against service users' need and readiness to engage with other services. Participants appreciated not feeling under undue pressure and recounted how the right amount of encouragement supported them to engage and make changes at their own pace. Narratives indicated service users experienced a considerable degree of control over their onward referral which helped them feel that when they did engage with other services, they were ready to do so:

> *There's no pressure or anything. If you can't do it, they don't ask why, they just say, "Oh, we'll try to sort something else out for you," ... She said, "Well, it needs to come from you." ... if it was, like, a sort of forced thing I think I would have problems going [to self-help organisation] … I hate it if I'm made to go.* (P11, Male, 45-49)

> *I certainly felt better from having spoken to her [Link Worker], and for her inspiring me to … kick me along and say, "Yes, you can do that, you can do that." And when I described the [negative] feelings I had she turned them around to be something else that made me feel loads better.* (P5, Female, 65-69)

The level of support that some service users required in order to engage with services, particularly those involving physical activity, appeared to be considerable. Link workers paced the level of support they offered, particularly in the initial stages:

For peer review only - http://bmjopen.bmj.com/site/about/guidelines.xhtml

> *I just expected the Link Worker to introduce me to the gym, and that would have been it. And I think, if it had just been [that] I would have turned round, and I would have gone the opposite direction. But because of the way it was so gradually and really professionally linked in to different things, I just felt as though I'd floated into it, rather than getting shoved from behind. I just felt as though I was gradually moved into it*. (P2, Female, 70-74)

Building self-confidence, self-reliance and independence was another facet of the Link Workers' approach, managed through ongoing support and persistence in finding the right motivational tools for the individual, whilst conveying the need for personal responsibility and resilience. This enabled service users to make changes to their lives, engage with other organisations and manage their long-term conditions:

> *I mean, she's just someone who can make you do things… Not as in a bad way, she sort of, like, empowers you, for want of a better word, to do it, you know what I mean? She gives you, "… give it a go," and she'll explain … and if you don't go she doesn't get disappointed or anything like that, she just says, "Oh, right, well, we'll sort something else out for you." …I think the only person that can help is myself, really. I think it's 99% me and the 1% of help I get from the rest of the people, but … they can only like, give you advice and encouragement, the rest has got to be yourself.* (P11, Male, 45-49)

The holistic service offered by the Link Workers was contrasted favourably with what was available or possible through the GP or with previous attempts by other health professionals to deal with complex health issues:

> *I couldn't go to my doctors and ask them, I couldn't phone the nurse and ask the nurse.* (P13, Male, 60-64)

> *It hasn't worked miracles, but it's nice that she's there and sometimes you don't bother your GP or whatever, you know. It's a brilliant service, I think.* (P26, Male, 70-74)

> *At the very beginning, we agreed that she'd work around me, not me around her, to work on the foods that I thought were suitable for me … I met up with dieticians before … I felt they were dictating the food I should have, not the food I wanted to have. So that's what surprised me about Ways to Wellness, they're working around me … it's different. You talk about it. You're relaxed … You're in charge of it.* (P6, Male, 65-69)

For peer review only - http://bmjopen.bmj.com/site/about/guidelines.xhtml

Positive impact of the Link worker social prescribing programme

Health-related behaviours

Many participants attended physical activity classes specifically tailored to their health needs, helping them to incrementally build confidence and strength to improve their physical health. Some participants noted that as a result of the Ways to Wellness programme, their level of fitness had improved:

> *I really enjoyed it … When I first started off, I was doing five minutes on the cycling machine and … I had that up to 20 minutes … I was up to 15 minutes on [the cross trainer] and I was pulling weights … they asked us all these relevant questions because of my medical history so I hadn't to do too much for my heart … I just feel a hell of a lot fitter.* (P28, Male, 70-74)

Participants who received healthy eating advice and cooking skills recounted in some detail how they had reduced their intake of unhealthy food and how they were equipped with the practical skills to incorporate healthier foods into their diet:

> *Certainly the* [name of course], *because that was a six week course…The first session was salad and pizza, then we did stir fry, soup last week. Next week it's going to be fish, then I think it's desserts on the sixth week. Brilliant. It's not boring, but I just can't believe how good I've found it. It's not just for cooking. … it's what you should be eating, it's the portion size which is so important.* (P14, Male, 60-64)

> *I was asked to do a food diary. You see straightaway that what you're doing is wrong. I think that was one of the biggest things why I lost quite a bit of weight, to be honest … you don't realise at the time, but it was nothing for me to eat eight slices of bread a day. That's where all the badness is. Now, I only have pitta bread, really, or a small brown sliced bread. That was a big help, with the directions pointing to the food … it's smaller portions and more variety as well … now, I eat more fruit in the mornings and in the afternoons, and I don't pick as much.* (P29, male, aged 60-64)

Participants described how these diet and physical activity lifestyle changes improved their physical health. Many noted weight reduction and increased energy and fitness that enabled them to break a cycle of illness and re-start activities, such as walking, shopping and gardening, which previously they felt unable to manage:

For peer review only - http://bmjopen.bmj.com/site/about/guidelines.xhtml

> *At one time, when I went up [a hill]… I used to stop … but now I can walk directly up … I can carry my shopping bags now … I'd get up and dance … [without feeling] I needed to sit down because I was out of breath.* (P2, Female, 70-74)

Mental health

Social isolation, low mood, anxiety and depression were commonly experienced. For some, this was related primarily to physical health, but for others, this was compounded by life events such as bereavement. Offering opportunities for activities, which allowed people to meet and socialise in the community, reduced social isolation and positively impacted on self-confidence, self-esteem and mental wellbeing:

> *After [partner] passed away I was, not a recluse, but I just didn't want to talk to anybody. But since I've been coming to see [the Link Worker] I've broadened my horizons and I get out … I've got a lot more confidence.* (P10, Male, 60-64)

> *Before the Ways to Wellness, I used to just stay in. But they explained that if you get out and about, it helps you, which has proven right … there were times when I wasn't feeling too good, and I thought, "I can't be bothered," but I've pushed myself. By the time I've got out and got back, I've felt 100% better … my health has changed a lot … it's made me feel a lot more confident that there's somebody there.* (P4, Female, 55-59)

Long-term health problems were linked to a range of social and economic factors including relationship breakdown, job loss, income reduction, debt and housing problems. Link Worker consultations noted socio-economic factors and, where possible, took practical action to maximise income, reduce debt and assist service users navigating the welfare benefits system:

> *Whatever money I owed like electricity and TV licence was in my mind always eating me from inside. I sorted out that and it just changed so many things … It changed my attitude, it changed my behaviour and it changed my mood … I am not depressed like before … I feel better about everything … I go out almost every day … I play more, I write, I do jobs at home … I read more … yes, a big change.* (P30, Female, 45-49)

> *Because of my language barrier some things I could not sort things out so easily, but she could. Even though I didn't know about Attendance Allowance she applied for that … I didn't know which benefit I could get. She said, "I can try this one." She tried it and she was successful and it helped me a lot.* (P16, Female, 65-69)

For peer review only - http://bmjopen.bmj.com/site/about/guidelines.xhtml

For those of working age, health-related unemployment was a major problem. Steps to assist with finding paid or volunteer work, returning people to work or having reasonable adjustments to work settings were undertaken and highly appreciated:

> *I felt 100% better after talking to [Link Worker], she was just excellent … She put us in contact with people [to] do a new CV and look for a new job, [and] found information out for us … which has given me the confidence to go back to my company … things were put in place at work, so that I could take my breaks when I needed them … It made a massive difference to me, personally … I was getting on board with [mental health condition], and now, I'm at work with renewed vigour.* (P9, Male, 55-59)

Long-term condition management

Weight reduction and increased fitness helped participants manage the pain and tiredness experienced as a result of their long-term health conditions. Some participants with diabetes noted being in better control of their condition due to reduced cholesterol and sugar levels. Accounts of positive improvements often followed years of worsening health and poor long-term condition management:

> *The instructor here has given me a programme that I'm working on to help build up the muscles for the legs and for my back … I'm managing [my arthritis] with the help of the gym … [also] my sugar levels … [have] come down to 4.9 … [and] my cholesterol was 3.1, which is good … all that had to do with Ways to Wellness and the exercise I've been doing.* (P6, Male, 65-69)

Long-term condition management services that service users were directed to by Link Workers were highlighted as extremely helpful, particularly the combination of expert and peer-led advice on coping and symptom management strategies:

> *The [name] group was wonderful because there were people there who had similar conditions … and they have medical professionals come in to talk about things that are specific to people with chest complaints … coping mechanisms … gentle exercises and things to help. It improves your stamina, your breathing abilities and everything. So yes, that has been absolutely wonderful … honestly it was the turning point in my life. Because I had reached rock bottom and I just needed practical help to try and get back up … you have to manage your expectations as well as your illness and it has helped considerably.* (P8, Female, 55-59)

For peer review only - http://bmjopen.bmj.com/site/about/guidelines.xhtml

Regularly engaging with services was challenging, particularly for people whose condition fluctuated and those suffering from more than one health problem. Service users worried about not always being able to attend, which was often a reason for not sustaining engagement with services in the past:

> *I was really down, so I couldn't go on Friday. It's been like that since I first joined. I've been missing [sessions] because of my COPD … bad turns … chest infections … [but] they [Ways to Wellness] restarted me again, so they've looked after me … they've been really, really good … they've stuck with me.* (P1, Male, 70-74)

Participants experienced Ways to Wellness as an approach which recognised the long-term and complex nature of their health problems. It supported realistic, progressive and personalised goal-setting. Participants' expectations of progress were therefore achievable and reflected that a long-term approach was necessary to make improvements, helping people to live with their conditions and improve their wellbeing:

> *I am on the road but it is slow … work in progress … if it was easy I would have done it years ago … I have been well impressed [with Ways to Wellness]… because they have a very practical approach and know that it has got to be incremental … you can't do everything at once so you have got to start small and build up … they have got the big picture in mind but lots of little steps in between to get there … I would like to get back to being wonderful in the next five minutes it is not going to happen. So I know that I have got to take it bit by bit so it sticks.* (P8, Female, 55-59*)*

> *[Link Worker] epitomises the word, wellbeing, because that's what she's got in mind for you, your wellbeing. Nothing else, she gives the impression that you're the only one that she's looking after, at that time, in that moment in time, you're the only one that matters to her.* (P9, Male, 55-59)

DISCUSSION

This study provides evidence about the impact of a Link Worker social prescribing programme on health and wellbeing via improvements in health-related behaviours and long-term condition management. The specific model of social prescribing examined here

For peer review only - http://bmjopen.bmj.com/site/about/guidelines.xhtml

comprised personalised support together with linkage into appropriate services facilitated by a Link Worker. The data reveal that key elements of the model are that it is: long-term in nature; addresses the co-existence of multi-morbidity, mental health problems and social isolation; and, where applicable, tackles related socio-economic issues. This study demonstrates that the rapport and quality of the relationship between the Link Worker and service user was central to achieving wellbeing, as well as key to successfully linking service users into a wide range of community, voluntary and NHS services identified as relevant to their situation Change in health-related behaviour and long-term condition management was facilitated through the use of setting realistic, progressive and personalised goals, problem solving, receiving regular feedback and social support. These behaviour change techniques have been shown to be effective in other lifestyle interventions. [23-25] Crucially these techniques were adopted whilst also supporting individuals to address social and economic problems. The Link Worker social prescribing programme engendered feelings of control and self-confidence, reduced social isolation and led to positive physical changes such as weight loss, increased physical activity, improved long-term condition management and mental health, greater resilience and effective coping strategies to manage relapses.

Psychosocial problems, particularly mental health conditions and social isolation, are the most common reasons for referral into social prescribing programmes. [4 6] A review exploring the social prescribing evidence base identified 24 studies diverse in methodology and the service being evaluated. [6] The quantitative evidence in the review was limited with the exception of one randomised controlled trial demonstrating clinically important benefits in managing psychosocial needs at four months. [26] The quality of eight studies using quantitative methods was poor, with small samples and high drop-out rates.[6] Qualitative evidence, from this review and more broadly, also varies in quality, but generally indicates that those referred into social prescribing schemes report improved mental wellbeing and

For peer review only - http://bmjopen.bmj.com/site/about/guidelines.xhtml

reduced social isolation. [6 9 27] A systematic review and meta-analysis of exercise referral schemes, a type of social prescribing intervention, targeted at sedentary individuals with or without a medical diagnosis was inconclusive with regards to increasing physical activity, although the heterogeneity of the quality and the nature of the schemes is likely to have contributed to this conclusion.[28] The data presented in our study provide a much more in-depth account of *how* Link Worker social prescribing positively influences long-term condition management and how this programme can lead to improved physical and mental health outcomes.

Strengths, limitations and implications

The strength of this work lies in the depth and consistency of the service user accounts regarding the impact of Link Worker social prescribing. Methodologically, we followed accepted practice in fieldwork, analysis and interpretation. [29 30] There are a number of limitations. Firstly, although the sample did include people with a wide range of long-term conditions, almost all with multi-morbidity, and many experiencing social isolation and mental health problems we did only interview people who agreed to a referral, then enrolled into and remained with Ways to Wellness. We therefore cannot make claims about patients with long-term conditions who did not engage in the first place or who dropped out of the programme early. Secondly, we cannot be certain of the extent to which the experiences of the 30 study participants reflect those of the 1,603 who used the service, although maximum variation sampling was used to include a wide range of participant experience. Thirdly, the study lacks precise data about levels of participation in 'linked' services. We therefore cannot draw specific conclusions about the effects on health and wellbeing with respect to intensity and duration of 'linked' service use. These areas are undoubtedly important for further research.

For peer review only - http://bmjopen.bmj.com/site/about/guidelines.xhtml

Long-term health conditions are arguably the greatest challenge facing the NHS; they have an adverse effect on quality of life and reduce life expectancy. Multi-morbidity accounts for over 70 percent of total healthcare spend. [31] Non-medical interventions are proposed as a cost-effective alternative to foster self-management in people with long-term conditions. [32 33] People from lower socio-economic groups experience higher levels of chronic disease, and also have poorer condition management, worse health outcomes, and higher mortality. [31] Behavioural risk factors for long-term conditions include poor diet, smoking and physical inactivity, all of which are socioeconomically patterned. [34] However, lifestyle behaviours are also affected by wider health determinants and are not simply choices that individuals make. [35 36] Supporting people to make meaningful and sustainable lifestyle changes is complex, time consuming, and a challenge for time-limited GPs. [11] Given the complex and long-term nature of the problems faced by service users, it is unsurprising that a holistic and relatively intensive approach, as offered by Ways to Wellness, is required in order to facilitate and maintain behaviour change, and it is clearly not possible, nor appropriate to offer this level of support routinely in primary care. Referral into social prescribing programmes is likely to continue due to the need to tackle long-term health conditions, multi-morbidity and an ageing population in the face of stretched NHS resources. [33 37] The range of approaches to, and models of, social prescribing [38] make it challenging to amass good quality evidence to inform commissioning. [37 38] In order to inform practitioners and commissioners, future research should be robust and comparative, addressing when, for whom and how well the programme works, including validated measures of intervention impact and accurate costs. [37] To this we would add further qualitative, particularly ethnographic, research to examine potential wider impacts on families and communities and how community-based, non-medical and personalised interventions supplement health care.

For peer review only - http://bmjopen.bmj.com/site/about/guidelines.xhtml

Acknowledgements

We thank the participants for sharing their experiences, Link Workers and Link Worker Managers at Mental Health Concern, First Contact Clinical, Healthworks and Changing Lives for their assistance with recruitment, and Chris Drinkwater, Tara Case and Amie Callan from Ways to Wellness for their support.

Contributors

SM, LP and NO'B conceived and designed the research and obtained funding. MS and SL undertook interviews. SM, MS, LP and SL contributed to the data analysis. SM, MS, SL, LP and NO'B wrote the paper. All authors read and approved the final version.

Funding

This work was funded by the Cabinet Office of the UK Government (grant number A1543). The researchers are independent of the funders and the views and opinions expressed in this paper are those of the authors and not necessarily those of the Cabinet Office. The funders and sponsors (Newcastle University) had no role in the design, collection, analysis and interpretation of the data, or the writing of this paper or the decision to submit this article for publications. All authors had full access to the data in the study and can take full responsibility for the integrity of the data and the accuracy of the analysis.

Competing interests

None

For peer review only - http://bmjopen.bmj.com/site/about/guidelines.xhtml

Ethics approval

This study was approved by Newcastle University Faculty of Medical Sciences Research Ethics Committee (00868/2014)

Data sharing statement

No additional data are available

For peer review only

For peer review only - http://bmjopen.bmj.com/site/about/guidelines.xhtml

References

1. Local Government Association. Just what the doctor ordered. Social Prescribing - a guide for local authorities. http://wwwlocalgovuk/documents/10180/7632544/L16-108+Just+what+the+doctor+ordered+-+social+prescribing+-+a+guide+to+local+authorities/f68612fc-0f86-4d25-aa23-56f4af33671d, 2016 (accessed 6 January 2017).

2. Social Prescribing Network. Report of the Annual Social Prescribing Network Conference. London: University of Westminster, 2016.

3. South J, Higgins TJ. Can social prescribing provide the missing link? *Primary Health Care Research and Development* 2008;9:310 - 18.

4. Mossabir R, Morris R, Kennedy A, et al. A scoping review to understand the effectiveness of linking schemes from healthcare providers to community resources to improve the health and well-being of people with long-term conditions. *Health & Social Care in the Community* 2015;23(5):467-84. doi: 10.1111/hsc.12176

5. Popay J, Kowarzik U, Mallinson S, et al. Social problems, primary care and pathways to help and support: addressing health inequalities at the individual level. part I: the GP perspective. *Journal of Epidemiology and Community Health* 2007;61(11):966-71. doi: 10.1136/jech.2007.061937

6. Kilgarriff-Foster A, O'Cathain A. Exploring the components and impact of social prescribing. *Journal of Public Mental Health* 2015;14(3):127-34.

7. Kimberlee R. What is the value of social prescribing? *Advances in Social Sciences Research Journal* 2016;3(3):29-35.

8. Dayson C, Bashir N. The social and economic impact of the Rotherham Social Prescribing Pilot. Centre for Regional Economic and Social Research, Sheffield Hallam

For peer review only

University https://www4.shu.ac.uk/research/cresr/sites/shu.ac.uk/files/social-economic-impact-rotherham.pdf, 2014 (accessed 10 January 2017).

9. Kimberlee R, Ward R, Jones M, et al. Proving our values: Measuring the economic impact of Well Spring Healthy LIving Centre's Social Prescribing for low level mental health isues encountered by GP services. Bristol: University of the West of England, 2014.

10. Husk K, Blockley K, Lovell R, et al. What approaches to social prescribing work, for whom, and in what circumstances? A protocol for a realist review. *Systematic reviews* 2016;5(1):93-93. doi: 10.1186/s13643-016-0269-6

11. Brandling J, House W. Social prescribing in general practice: adding meaning to medicine. *British Journal of General Practice* 2009;59(563):454-56. doi: 10.3399/bjgp09X421085

12. Ways to Wellness http://waystowellness.org.uk/ (accessed 14 September 2016).

13. NHS North East Innovation Fund. Year of Care, Thanks for the Petunias- a guide to developing and commissioning non-traditional providers to support the self management of people with long term conditions, 2011.

14. Department for Communities and Local Government. The English Indices of Deprivation 2010, 2013 https://www.gov.uk/government/statistics/english-indices-of-deprivation-2010 (accessed 4 Aug 2016).

15. Newcastle West Clinical Commissioning Group. Annual Report and Accounts 2013-14,. Newcastle, 2014.

16. Denzin NK, Lincoln YS. Handbook of qualitative research. London: Sage 2000.

17. Green J, Thorogood N. Qualitative Methods for Health Research. London: Sage 2014.

18. NVivo 10. Computer Assisted Software for Qualitative Analysis 2010 (accessed 20 July 2012).

19. Glaser B, Strauss A. The Discovery of Grounded Theory. Chicago: Aldine 1967.

For peer review only

20. Silverman D. Doing qualitative research. London: Sage 2000.

21. Barbour RS. Checklists for improving rigour in qualitative research: a case of the tail wagging the dog? *BMJ : British Medical Journal* 2001;322(7294):1115-17.

22. ONS. SOC2010 volume 3: the National Statistics Socio-economic classification (NS-SEC rebased on SOC2010) 2010 [Available from: http://webarchive.nationalarchives.gov.uk/20160105160709/http://www.ons.gov.uk/ons/guide-method/classifications/current-standard-classifications/soc2010/soc2010-volume-3-ns-sec--rebased-on-soc2010--user-manual/index.html (accessed 14 November 2014).

23. Greaves C, Sheppard K, Abraham C, et al. Systematic review of reviews of intervention components associated with increased effectiveness in dietary and physical activity interventions. *BMC Public Health* 2011;11(1):119.

24. Lara J, Evans EH, O'Brien N, et al. Association of behaviour change techniques with effectiveness of dietary interventions among adults of retirement age: a systematic review and meta-analysis of randomised controlled trials. *BMC Medicine* 2014;12 doi: 10.1186/s12916-014-0177-3

25. O'Brien N, McDonald S, Araujo-Soares V, et al. The features of interventions associated with long-term effectiveness of physical activity interventions in adults aged 55-70 years: a systematic review and meta-analysis. *Health Psychology Review* 2015;9(4):417-33. doi: 10.1080/17437199.2015.1012177

26. Grant C, Goodenough T, Harvey I, et al. A randomised controlled trial and economic evaluation of a referrals facilitator between primary care and the voluntary sector. *BMJ* 2000;320:419-23.

For peer review only - http://bmjopen.bmj.com/site/about/guidelines.xhtml

27. City and Hackney Clinical Commissioning Group and University of East London. Shine 2014 final report: Social prescribing: integrating GP and community assets for health, 2015.

28. Pavay TG, Taylor AH, Fox KR, et al. Effect of exercise referral schemes in primary care on physical activity and improving health outcomes: systematic review and meta-analysis. *BMJ* 2011;343:d6462.

29. Barbour R, S. The Newfound Credibility of Qualitative Research? Tales of Technical Essentialism and Co-Option. *Qualitative Health Research* 2003;13(7):1019-27.

30. Ritchie J, Lewis J. Qualitative Research Practice. A Guide for Social Scientists. London: Sage 2003.

31. Barnett K, Mercer SW, Norbury M, et al. Epidemiology of multimorbidity and implications for health care, research, and medical education: a cross-sectional study. *The Lancet* 2012;380(9836):37-43.

32. Department of Health. Our health, Our Care, Our Say: A New Direction for Community Services. 2006 http://www.dh.gov.uk/en/Publicationsandstatistics/Publications/PublicationsPolicyAndGuidance/DH_4127453 (accessed March 2015) 33. Coulter A, Roberts S, A. D. Delivering better services for people with long-term conditions: building the house of care. London: The King's Fund, 2013. https://www.kingsfund.org.uk/sites/files/kf/field/field_publication_file/delivering-better-services-for-people-with-long-term-conditions.pdf (accessed 14 Nov 2016).

34. Wardle J, Steptoe A. Socioeconomic differences in attitudes and beliefs about healthy lifestyles. *Journal of Epidemiology and Community Health* 2003;57(6):440-43. doi: 10.1136/jech.57.6.440

For peer review only - http://bmjopen.bmj.com/site/about/guidelines.xhtml

35. UCL Institute of Health Equity. Tackling Health Inequalities Through Action on the Social Determinants of Health http://www.instituteofhealthequity.org/projects/tackling-health-inequalities-through-action-on-the-social-determinants-of-health-lessons-from-experience 2014 (accessed 8 January 2015).

36. Salmon P, Hall GM. Patient empowerment and control: a psychological discourse in the service of medicine. *Social Science & Medicine,* 2003;57:1969-80.

37. Centre for Reviews and Dissemination. Evidence to inform the commissioning of social prescribing. York: The University of York, https://www.york.ac.uk/media/crd/Ev%20briefing_social_prescribing.pdf, 2015 (accessed 4 August 2016).

38. Kimberlee R. What is social prescribing? *Advances in Social Sciences Research Journal* 2015;2(102-110)

For peer review only

Coreq Checklist for Manuscript ID bmjopen-2016-015203 entitled "Social prescribing to improve health and wellbeing for people with long-term conditions: qualitative study of service user perceptions"

**Domain 1: Research team and reflexivity**			**Authors' response**
1.	Interviewer/facilitator	Which author/s conducted the interview or focus group?	Page 9, MS and SL conducted interviews
2.	Credentials	What were the researcher's credentials? *E.g. PhD, MD*	Page 1<br>SM PhD<br>MS PhD<br>SL BA (Hons)<br>LP PhD<br>NOB PhD
3.	Occupation	What was their occupation at the time of the study?	SM Senior Lecturer<br>MS Research Associate<br>SL Research Associate<br>LP Research Associate<br>NOB Research Methodologist
4.	Gender	Was the researcher male or female?	Page 1 Female
5.	Experience and training	What experience or training did the researcher have?	MS PhD training<br>SL extensive undergrad and post grad experience

For peer review only

Relationship with participants			
6.	Relationship established	Was a relationship established prior to study commencement?	Pages 8-9<br><br>No
7.	Participant knowledge of the interviewer	What did the participants know about the researcher? e.g. *personal goals, reasons for doing the research*	Pages 8-9<br><br>Reasons for doing the research
8.	Interviewer characteristics	What characteristics were reported about the interviewer/facilitator? e.g. *Bias, assumptions, reasons and interests in the research topic*	Pages 8-9<br><br>Interest in the impact of the intervention
**Domain 2: study design**			
Theoretical framework			
9.	Methodological orientation and Theory	What methodological orientation was stated to underpin the study? *e.g. grounded theory, discourse analysis, ethnography, phenomenology, content analysis*	Page 9<br><br>Thematic analysis
Participant selection			

For peer review only

10.	Sampling	How were participants selected? *e.g. purposive, convenience, consecutive, snowball*	Page 8<br><br>Purposive
11.	Method of approach	How were participants approached? e.g. *face-to-face, telephone, mail, email*	Page 8<br><br>Participants were approached initially by gatekeepers (Link Workers). Once willing to participate, researchers contacted participants by telephone
12.	Sample size	How many participants were in the study?	Page 10<br><br>N=30
13.	Non-participation	How many people refused to participate or dropped out? Reasons?	Page 8<br><br>As we used gatekeepers to contact participants, we do not have accurate numbers of how many people refused to participate, but anecdotally, most who were approached were willing to take part.<br><br>Following agreement to take part in the study, no participants dropped out.
Setting			
14.	Setting of data collection	Where was the data collected? e.g. *home, clinic, workplace*	Page 8-9<br><br>Most interviews took place in participants' homes, but some took place at a community venue.

For peer review only

15.	Presence of non-participants	Was anyone else present besides the participants and researchers?	No
16.	Description of sample	What are the important characteristics of the sample? *e.g. demographic data, date*	Page 11, Table 1
Data collection			
17.	Interview guide	Were questions, prompts, guides provided by the authors? Was it pilot tested?	Page 8.<br><br>Topic guide developed with prompts on: referral procedures; level and type of engagement with Ways to Wellness; goal-setting; linkage to other services; long-term condition management; changes resulting from involvement with Ways to Wellness; and views of the service.<br><br>Not pilot tested with participants.
18.	Repeat interviews	Were repeat interviews carried out? If yes, how many?	No
19.	Audio/visual recording	Did the research use audio or visual recording to collect the data?	Page 9<br><br>Data were digitally recorded
20.	Field notes	Were field notes made during and/or after the	Page 9

For peer review only

		interview or focus group?	Fieldnotes were made immediately after each interview and shared amongst the team.
21.	Duration	What was the duration of the interviews or focus group?	Page 9<br><br>Duration ranged between eight minutes and one hour 27 minutes (average 41 minutes)
22.	Data saturation	Was data saturation discussed?	Page 9<br><br>Interviews continued until data saturation was reached.
23.	Transcripts returned	Were transcripts returned to participants for comment and/or correction?	No
**Domain 3: analysis and findings**z			
Data analysis			
24.	Number of data coders	How many data coders coded the data?	Page 9<br><br>Two coders (MS, SL)
25.	Description of the coding tree	Did authors provide a description of the coding tree?	No
26.	Derivation of themes	Were themes identified in advance or derived from the	Page 9<br><br>Some a priori themes based on topic guide

For peer review only

		data?	identified in advance, but further themes emerged from the data
27.	Software	What software, if applicable, was used to manage the data?	Page 9<br><br>NVIVO
28.	Participant checking	Did participants provide feedback on the findings?	No
Reporting			
29.	Quotations presented	Were participant quotations presented to illustrate the themes / findings? Was each quotation identified? e.g. *participant number*	Pages 14-20<br><br>Participant quotes presented to illustrate themes, all participants identified by number, gender and age.
30.	Data and findings consistent	Was there consistency between the data presented and the findings?	Pages 14-20 (results) and Page 20-21 discussion<br><br>Yes
31.	Clarity of major themes	Were major themes clearly presented in the findings?	Pages 14-20<br><br>Major themes presented as:<br><br>Impact of long-term conditions and multi-morbidity<br><br>Link Worker Roles<br><br>Positive Impact of the Intervention
32.	Clarity of minor	Is there a description of diverse cases or	Page 22

For peer review only

	themes	discussion of minor themes?	Participants accounts were highly consistent, although data interrogated for ‘diverse/deviant’ cases.

For peer review only
